# Tumour compartment transcriptomics demonstrates the activation of inflammatory and odontogenic programmes in human adamantinomatous craniopharyngioma and identifies the MAPK/ERK pathway as a novel therapeutic target

**DOI:** 10.1007/s00401-018-1830-2

**Published:** 2018-03-14

**Authors:** John R. Apps, Gabriela Carreno, Jose Mario Gonzalez-Meljem, Scott Haston, Romain Guiho, Julie E. Cooper, Saba Manshaei, Nital Jani, Annett Hölsken, Benedetta Pettorini, Robert J. Beynon, Deborah M. Simpson, Helen C. Fraser, Ying Hong, Shirleen Hallang, Thomas J. Stone, Alex Virasami, Andrew M. Donson, David Jones, Kristian Aquilina, Helen Spoudeas, Abhijit R. Joshi, Richard Grundy, Lisa C. D. Storer, Márta Korbonits, David A. Hilton, Kyoko Tossell, Selvam Thavaraj, Mark A. Ungless, Jesus Gil, Rolf Buslei, Todd Hankinson, Darren Hargrave, Colin Goding, Cynthia L. Andoniadou, Paul Brogan, Thomas S. Jacques, Hywel J. Williams, Juan Pedro Martinez-Barbera

**Affiliations:** 1 Developmental Biology and Cancer Programme, Birth Defects Research Centre, UCL Great Ormond Street Institute of Child Health, University College London, London, UK; 2 Histopathology Department, Great Ormond Street Hospital NHS Trust, London, UK; 3 Basic Research Department, National Institute of Geriatrics, Mexico City, Mexico; 4 Centre for Translational Omics-GOSgene, Genetics and Genomic Medicine Programme, UCL Institute of Child Health, University College London, London, UK; 5 Department of Neuropathology, Friedrich-Alexander University Erlangen-Nürnberg (FAU), Erlangen, Germany; 6 Alder Hey Children’s Hospital NHS Foundation Trust, Liverpool, UK; 7 Centre for Proteome Research, Institute of Integrative Biology, University of Liverpool, Liverpool, UK; 8 Infection, Immunity and Inflammation Programme, UCL Great Ormond Street Institute of Child Health, University College London, London, UK; 9 Centre for Craniofacial and Regenerative Biology, King’s College London, London, UK; 10 Department of Pediatrics, University of Colorado Anschutz Medical Campus, Aurora, CO USA; 11 German Cancer Research Center (DKFZ), Heidelberg, Germany; 12 Neurosurgery Department, Great Ormond Street Hospital NHS Trust, London, UK; 13 Endocrinology Department, Great Ormond Street Hospital NHS Trust, London, UK; 14 Laboratory Medicine, Royal Victoria Infirmary, Newcastle, UK; 15 Children’s Brain Tumour Research Centre, University of Nottingham, Nottingham, UK; 16 William Harvey Research Institute, Barts and the London School of Medicine and Dentistry, Queen Mary University, London, UK; 17 Pathology Department, Plymouth Hospitals NHS Trust, Plymouth, UK; 18 MRC London Institute of Medical Sciences, Imperial College London, London, UK; 19 Head and Neck Pathology, Dental Institute, King’s College London, London, UK; 20 Institute of Pathology, Klinikum Sozialstiftung Bamberg, Bamberg, Germany; 21 Haematology and Oncology Department, Great Ormond Street Hospital NHS Trust, London, UK; 22 Ludwig Institute for Cancer Research, Oxford University, Old Road Campus, Headington, Oxford, UK; 23 Centre for Craniofacial and Regenerative Biology, King’s College London, Guy’s Hospital, Floor 27 Tower Wing, London, UK; 24 Department of Internal Medicine III, Technische Universität Dresden, Fetscherstaße 74, 01307 Dresden, Germany; 25 Rheumatology Department, Great Ormond Street Hospital NHS Trust, London, UK

**Keywords:** Craniopharyngioma, Odontogenesis, Inflammasome, IL1-β, MAPK/ERK pathway, Trametinib, Paracrine signalling

## Abstract

Adamantinomatous craniopharyngiomas (ACPs) are clinically challenging tumours, the majority of which have activating mutations in *CTNNB1*. They are histologically complex, showing cystic and solid components, the latter comprised of different morphological cell types (e.g. β-catenin-accumulating cluster cells and palisading epithelium), surrounded by a florid glial reaction with immune cells. Here, we have carried out RNA sequencing on 18 ACP samples and integrated these data with an existing ACP transcriptomic dataset. No studies so far have examined the patterns of gene expression within the different cellular compartments of the tumour. To achieve this goal, we have combined laser capture microdissection with computational analyses to reveal groups of genes that are associated with either epithelial tumour cells (clusters and palisading epithelium), glial tissue or immune infiltrate. We use these human ACP molecular signatures and RNA-Seq data from two ACP mouse models to reveal that cell clusters are molecularly analogous to the enamel knot, a critical signalling centre controlling normal tooth morphogenesis. Supporting this finding, we show that human cluster cells express high levels of several members of the FGF, TGFB and BMP families of secreted factors, which signal to neighbouring cells as evidenced by immunostaining against the phosphorylated proteins pERK1/2, pSMAD3 and pSMAD1/5/9 in both human and mouse ACP. We reveal that inhibiting the MAPK/ERK pathway with trametinib, a clinically approved MEK inhibitor, results in reduced proliferation and increased apoptosis in explant cultures of human and mouse ACP. Finally, we analyse a prominent molecular signature in the glial reactive tissue to characterise the inflammatory microenvironment and uncover the activation of inflammasomes in human ACP. We validate these results by immunostaining against immune cell markers, cytokine ELISA and proteome analysis in both solid tumour and cystic fluid from ACP patients. Our data support a new molecular paradigm for understanding ACP tumorigenesis as an aberrant mimic of natural tooth development and opens new therapeutic opportunities by revealing the activation of the MAPK/ERK and inflammasome pathways in human ACP.

**Electronic supplementary material:**

The online version of this article (10.1007/s00401-018-1830-2) contains supplementary material, which is available to authorized users.

## Introduction

Adamantinomatous craniopharyngioma (ACP) is the most common tumour of the sellar region in children [39]. Despite being cytologically benign, ACPs display clinically aggressive behaviour such as destruction of the pituitary gland and invasion of the hypothalamus and visual pathways, altogether leading to chronic severe morbidity and increased mortality during long-term follow-up [41, 43].

ACPs are histologically complex tumours with variable cystic, calcified and solid components. They frequently invade adjacent tissues with infiltrating finger-like structures surrounded by a florid glial and inflammatory reactive tissue [39]. A pathognomonic feature of ACP is the presence of anuclear ghost cells, also known as “wet keratin”, while other common features include calcification and cholesterol clefts [35, 39]. The majority of ACPs have somatic activating mutations in *CTNNB1*, the gene encoding β-catenin [8, 10, 31, 35, 50]. However, nucleo-cytoplasmic accumulation of β-catenin is limited to only a small proportion of cells, often correlating with epithelial whorl-like structures (referred to as β-catenin-accumulating cell clusters), or in single cells throughout the tumour [8, 23, 25, 31, 41]. As expected, these regions correlate with WNT pathway activation, evidenced by the expression of pathway target genes (e.g. *AXIN2* and *LEF1*) [25, 51]. In contrast, in other histological compartments such as the palisading epithelium or stellate reticulum, β-catenin expression is limited to the cell membrane and activation of the WNT pathway is less pronounced [25]. The similarities between ACP and both normal tooth development and odontogenic tumours have been recognised for decades, on the basis of their comparable histopathology and co-expression of enamel proteins, proteinases and some keratins, as well as the occasional identification of fully formed teeth in ACP [6, 42, 49]. However, the molecular mechanisms underlying these commonalities are not well understood [7, 10, 19, 29, 32, 42, 45, 51, 54].

Insight into the functional significance of the β-catenin-accumulating cell clusters has been provided by studies conducted in murine ACP models, where a degradation-resistant (activated) form of β-catenin is expressed in either Rathke’s pouch derivatives (*Hesx1*^*Cre/*+^*/Ctnnb1*^*lox(ex3)/*+^ mouse line; ACP embryonic model) or Sox2-expressing adult pituitary stem cells (*Sox2*^*CreERT2/*+^*/Ctnnb1*^*lox(ex3)/*+^ mouse line; ACP inducible model). Evidence gathered from both models suggests these clusters act in a paracrine manner, driving tumour growth and/or invasion into surrounding tissues through the secretion of a wide range of factors (e.g. SHH, FGFs, BMPs, TGFB1; as well as pro-inflammatory mediators such IL1, IL6 and other CXC and CC chemokines) [1, 2, 14, 17, 41, 53]. This hypothesis is consistent with their location at the leading edge of tissue invasion in human tumours [3, 9, 53]. These findings have raised interest in discovering therapeutic approaches targeting these clusters and highlighted a need to better understand the pathways and cellular processes active both within clusters and the responding tissues.

Two recent publications have analysed mRNA expression microarray profiles of ACPs, comparing either with other tumour types and control tissues, or with the *BRAF* mutation-driven papillary craniopharyngioma (PCP) subtype. These studies have highlighted the differences between ACP and PCP and identified a number of potential therapeutic targets using differential expression analysis, many of these previously anticipated by studies using the murine models [21, 26]. However, correlation between dysregulated gene pathways in human ACP and tumour architecture has been limited to the analyses of specific proteins by immunostaining. Revealing which pathways are dysregulated in specific tumour cellular compartments is important to increase our understanding of the pathogenesis of human ACP. Additionally, it is a necessary step to determine which cells are likely to be susceptible to specific targeted therapies, helping predict possible outcomes. For instance, pancreatic ductal cell carcinoma (PDCA) epithelial cells secrete SHH and activate the pathway in the tumours, but its targeted inhibition in mouse models and patients causes disruption of the host-derived stroma with little effect on the cancer epithelial cells, resulting in rapid disease progression and increased aggressiveness [36, 46].

In this manuscript, we have performed a comprehensive gene expression study of 18 human ACPs combining whole-tumour RNA-Seq with transcriptomics of laser capture microdissected tumour cellular components to reveal the molecular signatures of specific tumour cell compartments. We complement this gene expression study with proteome analysis and ELISA of both tumour and cystic fluid from ACP patients. Our data provide a molecular rationale for the resemblance of ACP and tooth development and highlight a complex signalling cascade orchestrated by the cluster cells. Of clinical relevance, we identify the MAPK/ERK pathway and inflammasome signalling as potentially targetable pathways and provide preclinical evidence supporting the use of MEK inhibitors against human ACP.

## Materials and methods

### Human tumour and pituitary samples

Anonymised archival frozen and formalin-fixed paraffin-embedded (FFPE) specimens of ACP and non-functioning pituitary adenoma (NFPA) were identified in the local pathology archive, through the Childhood’s Cancer and Leukaemia Group Tissue Bank, Brain UK and from collaborators. Fetal pituitary tissue (19 weeks corrected gestational age) was accessed through the Human Developmental Biology Resource (HDBR). Further details can be found in Suppl. Materials and Methods (Online Resource 1).

### Laser capture microdissection (LCM)

Two cases (JA004, JA029) were used for LCM because they contained easily definable histological features (clusters, palisading epithelium and glial reaction) and cryopreserved tissue of sufficient quality was available. Further details can be found in Suppl. Materials and Methods (Online Resource 1).

### RNA sequencing

For human ACP samples sequencing was performed by UCL Genomics. Murine ACP samples were sequenced by the Oxford Wellcome Trust Centre for Human Genetics. Further details can be found in Suppl. Materials and Methods (Online Resource 1).

### Immunostaining of histological sections

Immunohistochemistry and immunofluorescence staining was performed as previously described [1, 14, 26]. Antibody details can be found in Suppl. Materials and Methods (Online Resource 1).

### Ex vivo culture of mouse and human tumours

Ex vivo culture of neoplastic pituitaries was performed as described [17] in the presence of either trametinib at 2 or 20 nM (Mekinist) or DMSO (vehicle control). Histological analysis was performed after 18 h. Small pieces of human ACP (around 1–2 mm^3^) were cultured in identical conditions. The proportion of Ki67 positive and cleaved caspase-3 cells in the ex vivo culture experiments was calculated as an index out of the total DAPI-stained nuclei. Over 150,000 DAPI nuclei were counted from six histological sections per pituitary in a total of six neoplastic pituitaries. Three human tumours were used in the ex vivo experiments and the Ki-67 and cleaved caspase-3 index calculated as described above from four histological sections per tumour (over 192,000 cells counted).

### Cytokine multiplex ELISA

Levels of TNF-α, IFN-γ, IL-1β, IL-6. IL-8, IL-10 and IL-18 were measured in ACP tumour and ACP cystic fluid using a Meso Scale Discovery (MSD) multiplex kit (Meso Scale Diagnostics) as per the manufacturer’s instructions. For solid tumours, protein lysates were extracted from pieces of fresh frozen ACP. Briefly, 6- to 28-mg samples were placed in 250 μl of lysis buffer (150 mM NaCl, 20 mM Tris pH 7.5, 1 mM EDTA, 1 mM EGTA, 1% Triton, with Protease Inhibitor (Roche), 1 mM sodium orthovanadate and 25 mM sodium fluoride). Samples were sonicated on ice and left for 30 min at 4 °C, followed by centrifugation at 20,000*g* for 10 min and collection of supernatant. Protein concentrations were quantified by Bradford assay. Cytokine levels were normalised against total protein added to the ELISA.

## Results

### Samples and RNA sequencing data analysis

**Fig. 1:**
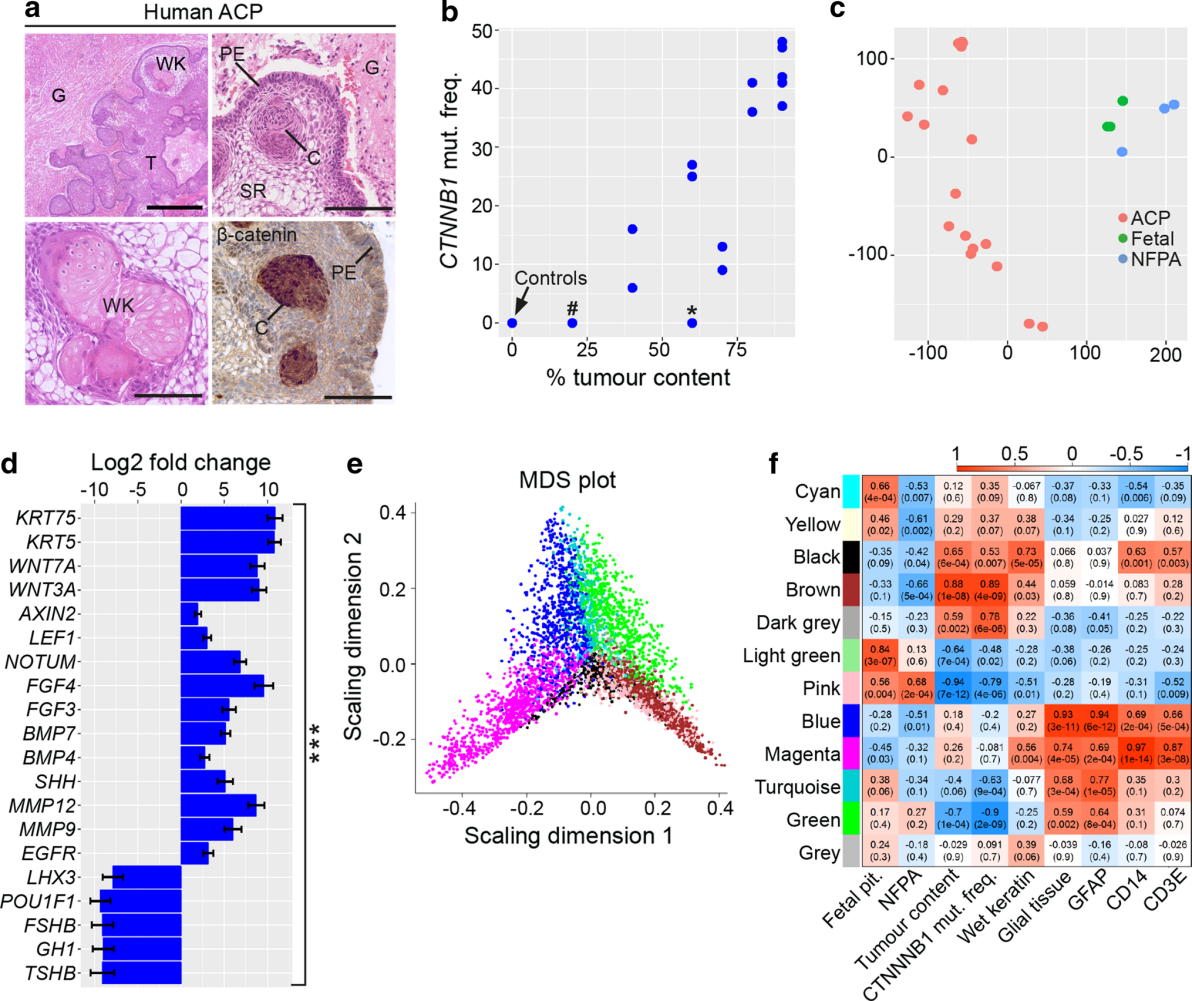
Bioinformatics analysis of gene expression profiling of human ACP whole tumours identifies modules of genes potentially associated with specific tumour cell compartments. **a** Representative histology of ACP samples showing and areas of tumour (T), reactive glial tissue (G), wet keratin/ghost cells (WK), epithelial whorls (C) (epithelial whorls referred to in this paper as clusters), palisading epithelium (PE) and stellate reticulum (SR). Immunohistochemistry using antibodies against β-catenin (β-cat) on case JA029 showing clusters of cells with nuclear-cytoplasmic accumulation. Scale bars 400 μm (top left panel), 100 μm (other three panels). **b** Scatterplot revealing a significant correlation between *CTNNB1* mutation frequency and histologically assessed tumour content. # case JA011; * case JA005; controls: fetal pituitaries and NFPA tissues. See Suppl. Table 1 (Online Resource 2) for sample details. **c** Principal component analysis plot showing the separation between adamantinomatous craniopharyngioma (ACP), non-functioning pituitary adenoma (NFPA) and control fetal pituitary tissues (fetal). **d** Bar plot of selected statistically significant and differentially expressed genes, as assessed by DESeq2, in ACP tumours compared with control fetal tissue. Higher than 0 means higher expression in ACP and lower than 0 means higher in control tissue. The most up-regulated genes in ACP tumours are keratins. Other up-regulated genes include *WNTs* and WNT pathway targets (e.g. *NOTUM*, *AXIN2*, *LEF1*), genes known to be expressed in ACP (e.g. *FGFs*, *BMPs*) and previously suggested therapeutic targets (*SHH*, *MMP12*, *MMP9*, *EGFR*). Pituitary transcription factors (*LHX3*, *POU1F1*) and pituitary hormones (e.g. *FSHB*, *GH1*, *TSHB*) are up-regulated in controls. See Suppl. Table 3 (Online Resource 5) for details. Error bars = 1 standard error, *** adjusted *p* value < 1 × 10^−9^. **e** Multidimensional scaling plot of expression patterns of the 5000 most differentially expressed genes included in the weighted gene co-expression network analysis (WGCNA) analysis. The colour of each gene indicates it membership to a co-expressed gene expression module. **f** Heatmap of correlations between each module’s gene expression profile and phenotypic information. Scale bar indicate *r* value − 1 to + 1. For instance, the brown module shows a strong correlation with tumour content and mutational frequency, whilst the blue module correlates with the presence of glial reactive tissue and GFAP

A total of 18 primary ACP samples and six control tissues (three fetal pituitaries and three non-functioning pituitary adenomas, NFPA) were analysed by RNA-Seq. We chose fetal pituitaries as control sample because ACP is thought to derive from remnants of Rathke’s pouch, the primordium of the anterior pituitary. RNA quality from post-mortem normal adult pituitaries was inadequate for RNA sequencing. As NFPA are tumours containing cells similar to the normal adult pituitary and are known to cluster with normal pituitary tissue on expression analysis [21], these were also used as controls. Activating mutations in exon 3 of *CTNNB1* were identified in all of the ACP cases, except for JA011 and JA005, with a mutation allele frequency ranging between 6% and 48% [Suppl. Table 1 (Online Resource 2)]. Targeted DNA sequencing of an adjacent frozen sample of case JA011 revealed a *CTNNB1* p.Gly34Arg mutation with a variant allele frequency of 4.7% [Suppl. Table 1 (Online Resource 2)]. There was insufficient material to perform targeted sequencing on JA005. Immunohistochemistry against β-catenin in case JA005 failed to identify cells, which accumulated the protein and analysis of RNA data did not reveal mutations in other *CTNNB1* exons [Suppl. Table 1 (Online Resource 2)]. The allelic frequencies of mutant *CTNNB1* significantly correlated with the estimated histological tumour content (*r* = 0.88, *p* = 6.61 × 10^−8^) and were consistent with the presence of a heterozygous mutation within all tumour cells [Suppl. Table 1 (Online Resource 2); Fig. 1b]. Principal component analysis (PCA) and clustering confirmed the separation of tumours (including JA005 and JA011) from controls [Fig. 1c; Suppl. Fig. 1a (Online Resource 3)]. Differential gene expression revealed that a total of 6099 genes were significantly expressed at higher levels in tumours versus controls, while 5211 genes were higher in controls versus tumours (adjusted *p* value < 0.1) [Suppl. Table 2a (Online Resource 4)]. The genes and ontology pathways found to be dysregulated in our dataset are consistent with published RNA and immunohistochemical expression studies and demonstrate that these results are robust and biologically meaningful [Fig. 1d; Suppl. Table 2a, b (Online Resource 4) and Suppl. Table 3 (Online Resource 5)] [1, 2, 10, 16, 21, 24].

### Computational modular analysis and transcriptomics of laser capture microdissected tumour cells reveal the molecular signatures of specific cellular compartments

Next, we aimed to further dissect the molecular signatures obtained from the RNA-Seq data and establish if these correlated with particular tumour characteristics including diagnosis, percentage of tumour content, *CTNNB1* mutation allele frequency and the presence of specific histological features. Weighted gene co-expression network analysis (WGCNA) was used to describe the correlation patterns among genes across samples, resulting in the identification of 12 distinct patterns of gene expression (modules), which were assigned a colour identifier (Fig. 1e). Three major distinct patterns of gene expression across the samples were determined: (1) the brown module, consisting of genes relating to epithelial differentiation and whose expression correlated with percentage tumour content and *CTNNB1* mutation allele frequency [Fig. 1f; Suppl. Table 4a, e (Online Resource 6); Suppl. Fig. 2a (Online Resource 3)]; (2) the blue and turquoise modules, containing genes involved in nervous system development, whose expression correlated with the presence of reactive glial tissue [Fig. 1f; Suppl. Table 4a, j, l (Online Resource 6); Suppl. Fig. 3a, b (Online Resource 3)]; (3) the magenta module, including inflammation-related genes whose expression correlated with immune cell markers such as CD14 [Fig. 1f; Suppl. Table 4a, k (online Resource 6); Suppl. Fig. 2d (Online Resource 3)]. Further details of other identified modules are presented in Suppl. Results (Online Resource 1), Suppl. Figs. 2–4 (Online Resource 3) and Suppl. Table 4 (Online Resource 6). WGCNA of the human ACP microarray data published by Gump et al. [21] also showed moderate preservation of the major modules with our datasets [Suppl. Results (Online Resource 1)], suggesting that the molecular signatures of the main cellular types within ACP samples (i.e. tumour epithelium, reactive glial tissue and inflammatory infiltrate) may be contained in specific modules.

**Fig. 2:**
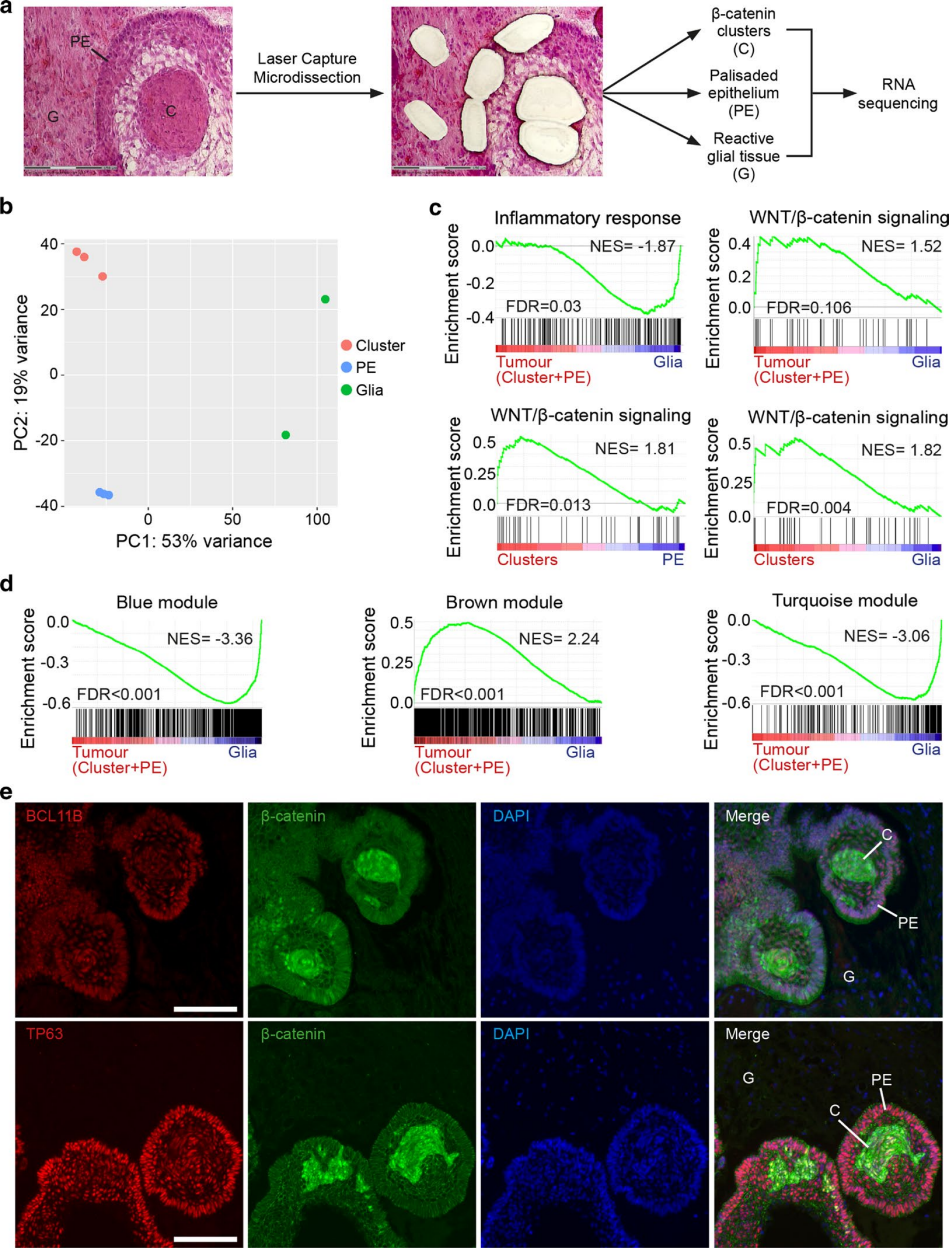
Gene profiling of laser capture microdissected tumour cells confirms the WGCNA, identifying modules associated with tumour cell compartments and revealing novel ACP genes. **a** Scheme of the experimental approach. Histological sections of tumour samples JA004 and JA029 were subjected to laser capture microdissection (LCM) to isolate β-catenin-accumulating cell clusters (C), palisading epithelium (PE) and glial reactive tissue (G). Biological duplicates were performed for clusters and palisading epithelium in case JA004. RNA was purified from each of these tumour cell compartments, amplified and sequenced. **b** Principal component analysis reveals grouping of the data from laser capture microdissected samples. **c** Gene set enrichment analysis revealing the enrichment of an inflammatory signature in microdissected glial reactive tissue relative to tumour tissue (i.e. genes included in clusters plus palisading epithelium), whilst a WNT signalling expression signature is associated with the microdissected tumour tissue. Enrichment for WNT signalling was stronger in the clusters relative to both PE and glial tissue. **d** Gene set enrichment analysis showing the enrichment of the brown module genes with a signature of tumour cell compartments (i.e. genes including in cluster cells plus palisading epithelium). In contrast, both the blue and turquoise module genes are predominantly expressed by reactive glia. **e** Double immunofluorescence staining revealing the expression of BCL11B and TP63 in the epithelial components of the tumour, including palisading epithelium (PE) and β-catenin accumulating clusters (C), but not in reactive glial tissue (G). NES normalised enrichment score, FDR false discovery rate. Scale bars 100 μm

To validate the WGCNA results, we profiled specific cellular compartments of human ACP, through the isolation of cluster cells, palisading epithelium and reactive glial tissue by laser capture microdissection (LCM) (Fig. 2a, b). As a result of the rarity of suitable tissue, we performed this experiment using only two human ACP tumours, nonetheless RNA-Seq data was robust and representative of the isolated cell compartments. For instance, gene set enrichment analysis (GSEA) confirmed the enrichment of a WNT signalling expression signature in tumour tissue (i.e. clusters plus palisading epithelium, PE) when compared with glial reactive tissue (normalised enrichment score (NES) = 1.52, false discovery rate (FDR) = 0.106), whilst an inflammatory response signature was associated with the glial reactive tissue (NES = − 1.87, FDR = 0.03) (Fig. 2c). Enrichment for WNT signalling was stronger in the clusters relative to both PE (NES = 1.81, FDR = 0.013) and glial tissue (NES = 1.82, FDR = 0.004), in agreement with the higher expression of WNT target genes in cluster cells (e.g. *AXIN2*, *LEF1* and *NOTUM*) (Fig. 2c) [14]. A full list of the differentially expressed genes between clusters, palisading epithelium and reactive glia is shown in Suppl. Table 2c–e (Online Resource 4).

By systematically comparing the molecular signatures obtained from these profiling experiments with the modules previously described, we confirmed that the brown module genes were enriched in the signature of tumour cell compartments (i.e. cluster cells and palisading epithelium) (NES = 2.24, FDR < 0.001) (Fig. 2d). In contrast, both the blue and turquoise module genes were predominantly expressed by reactive glia (blue, NES = − 3.36; turquoise, NES = − 3.06; FDR < 0.001) (Fig. 2d). Supporting the notion that these modules represent specific signatures, genes known to be expressed in tumour epithelium, such as WNT targets (e.g. *AXIN2*, *NOTUM*), *FGF3* and *BMP4* were contained in the brown module [Suppl. Table 4a (Online Resource 6)]. Likewise, genes known to be expressed in the glial reactive tissue, such as *GFAP*, *S100B* and *NKX2.2* were contained in the blue and turquoise modules [Suppl. Table 4a (Online Resource 6)]. Of interest, the three genes with the strongest brown module membership score (a measure of the degree of co-expression) were *TP63*, *APCDD1L* and *BCL11B* [Suppl. Table 3a (Online Resource 6)]. *TP63* has previously been shown to be expressed in the tumour cells in human ACP [11], but *APCDD1L* and *BCL11B* have not been implicated in ACP. Consistently, immunofluorescence of ACP histological sections revealed the expression of *BCL11B* and *TP63* exclusively in tumour cell compartments, including clusters, palisading epithelium and stellate reticulum, but not within surrounding reactive tissue (Fig. 2e). Similarly, immunofluorescence revealed expression of APCDD1L mostly in the cluster cells with no expression in the vast majority of the glial tissue except for cells adjacent to the tumour cells [Suppl. Fig. 5 (Online Resource 3)]. This is in agreement with the differential expression analysis of the laser capture microdissection dataset showing that *APCDD1L* is highly expressed in the clusters versus glial reactive tissue [40.46-fold, adjusted *p* value = 0.12; Suppl. Table 2e (Online Resource 4)]. Plotting of normalised expression levels confirmed the relationship of these brown module genes with *CTNNB1* mutation allele frequency, further supporting that expression of these genes is enriched within the tumour cells (*r* = 0.83, 0.95, 0.96 respectively for each gene, *p* < 1 × 10^−5^) [Suppl. Fig. 6 (Online Resource 3)]. These datasets represent a useful resource for the identification of novel genes specifically expressed or highly enriched within particular human ACP cell compartments. Together, these studies have revealed the molecular signatures of the tumour epithelium (i.e. cluster cells and palisading epithelium) and glial reactive tissue (astrocytes and immune cells).

### Human ACP clusters are molecularly analogous to the enamel knot and activate a transcriptional programme resembling odontogenesis

**Fig. 3:**
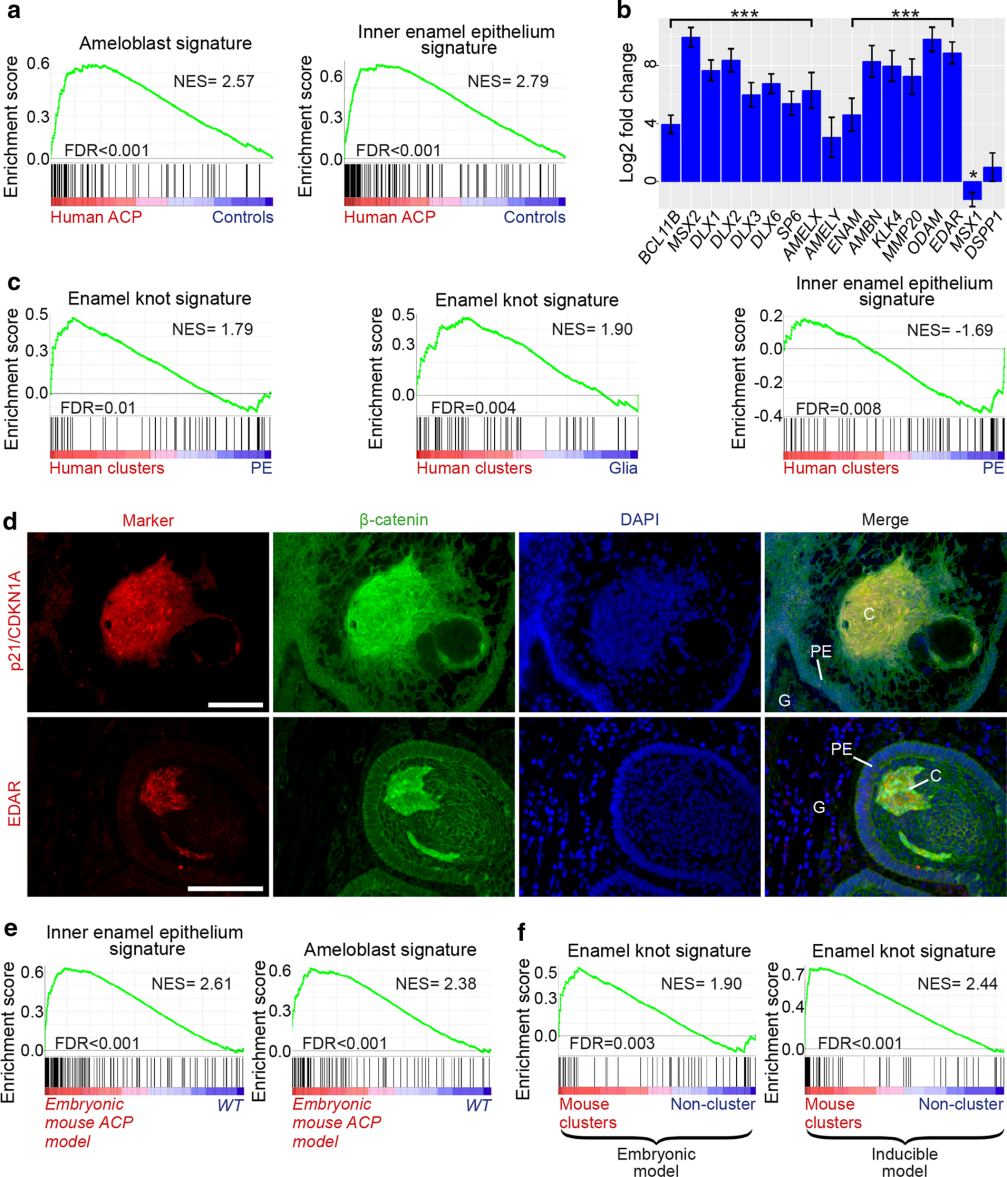
ACP and developing mammalian tooth share common molecular signatures. **a** Gene set enrichment plots showing that ACP tumours are enriched for genes expressed by ameloblasts and inner enamel epithelium. Results obtained from using the RNA dataset from profiling whole ACP tumours. **b** Expression of relevant ameloblast-related genes is significantly expressed at higher levels in whole ACP tumours compared with control tissues (fetal pituitary and NFPA). See Suppl. Table 5 (Online Resource 7) for details (*** adjusted *p* value < 1 × 10^−7^, * adjusted *p* value = 0.028). **c** Gene set enrichment plots showing that the cluster cells are enriched for genes expressed in the enamel knot whilst palisading epithelium shows a signature of inner enamel epithelia at cap stage. Results obtained from using the RNA dataset from profiling microdissected ACP compartments. **d** Double immunofluorescent staining reveals the co-expression of p21/CDKN1A and EDAR, two enamel knot markers, in the β-catenin-accumulating clusters. **e** Gene set enrichment analysis showing that both the inner enamel epithelium and ameloblast gene signatures are enriched in the embryonic mouse ACP model compared with wild-type controls (WT). **f** Enrichment plots confirming that mouse clusters from both the embryonic and inducible ACP mouse models show a molecular signature of the enamel knot. NES normalised enrichment score, FDR false discovery rate. Scale bars 100 μm

We then used the RNA data from profiling whole tumours and compartment-specific molecular signatures obtained by LCM to explore the relationship between ACP and odontogenesis at a molecular level. The brown module genes, enriched in the signature of tumour cell compartments, contained genes related to odontogenesis [Suppl. Table 4a, e (Online Resource 6]. GSEA was performed using sets of genes experimentally confirmed to be expressed in specific cell types during tooth development in human and other species [30]. These studies revealed that, compared to control tissues (fetal pituitary and NFPA), human ACP tumours (based on whole-tumour RNA datasets) were enriched for genes expressed in both ameloblasts (NES = 2.57, FDR < 0.001) and their precursors, the inner enamel epithelium (NES = 2.79, FDR < 0.001; dataset from all stages of inner epithelium development (Fig. 3a) [30]. The expression of ameloblast transcription factors (e.g. *BCL11B*, *MSX2*), enamel genes (*ENAM*, *AMELX*, *AMELY*, *AMBN*) and proteinases (*MMP20*, *KLK4*) was significantly higher in human ACP compared with control tissues [Fig. 3b; Suppl. Table 5 (Online Resource 7)]. In contrast, genes specifically expressed in dental mesenchyme-derived odontoblasts were not up-regulated in human ACP (e.g. *MSX1*, *DSPP*) [Fig. 3b; Suppl. Table 5 (Online Resource 7)].

By comparing the molecular signatures of specific tumour compartments obtained by LCM with curated gene sets derived from distinct cell types present during tooth development [30], we identified molecular similarities between the ACP β-catenin-accumulating cell clusters and the enamel knot, a critical signalling centre within the developing tooth [28, 55]. Gene set enrichment analysis revealed a significant enrichment of the enamel knot gene signature in human cluster cells when compared with either palisading epithelium (NES = 1.90, FDR = 0.004) or to reactive glial tissue (NES = 1.79, FDR = 0.01) (Fig. 3c). Enamel knot signals act on the inner enamel epithelium at the cap stage of tooth development to control proliferation and tooth morphogenesis, and we identified a strong enrichment between palisading epithelium and cap-stage enamel epithelium [30], suggesting that palisading and enamel epithelium may be equivalent structures (NES = − 1.69, FDR = 0.008) (Fig. 3c).

We validated these findings further by immunofluorescence against p21/CDKN1A, a marker of the enamel knot, which showed specific signal mostly restricted to human ACP clusters (*n* = 8 tumour samples; Fig. 3d), consistent with previous reports [10, 17]. In addition, the ectodysplasin receptor (EDAR), whose signalling pathway has been strongly implicated in enamel knot formation but not previously studied in ACP [55, 56], was highly expressed in ACP tumours compared with controls (467.88-fold; adjusted *p* value = 1.37E−29) and in β-catenin clusters relative to palisading epithelium (10.85-fold; adjusted *p* value = 0.0001) or glial reactive tissue (357.05-fold; adjusted *p* value = 9.77E−05) [Suppl. Table 2a, d, e (Online Resource 4)]. Immunofluorescence confirmed the expression of EDAR in human cluster cells and its absence in the glial reactive tissue, further confirming similarities between ACP clusters and the enamel knot (*n* = 5 human tumours; Fig. 3d).

To test further these findings, we performed RNA-Seq on three *Hesx1*^*Cre/*+^*/Ctnnb1*^*lox(ex3)/*+^ neoplastic and three control pituitaries at postnatal day 1 (P1). As in human ACP, GSEA also confirmed greater expression of inner enamel epithelium (cap stage) (NES = 2.61, FDR < 0.001) and ameloblast (NES = 2.38, FDR < 0.001) genes in *Hesx1*^*Cre/*+^*/Ctnnb1*^*lox(ex3)/*+^ neoplastic pituitaries at P1 compared with wild-type controls, further highlighting the molecular relationship between mouse ACP and tooth development [Fig. 3e; Suppl. Table 2f (Online Resource 4)]. Mouse ACP also contains β-catenin-accumulating cell clusters, and we have previously characterised their expression profiles in both the *Hesx1*^*Cre/*+^*/Ctnnb1*^*lox(ex3)/*+^ embryonic and the *Sox2*^*CreERT2/*+^*/Ctnnb1*^*lox(ex3)/*+^ inducible mouse models of ACP [1, 17]. GSEA revealed a molecular signature of the enamel knot in the clusters from both of these mouse models (embryonic model, NES = 1.90, FDR = 0.003; inducible model NES = 2.44, FDR < 0.001) (Fig. 3f). Expression of p21 has recently been reported to be enriched in the clusters in mouse ACP [17], but EDAR immunostaining was inconclusive, possibly because the antibody does not recognise mouse EDAR. Together, these results reveal molecular analogies shared between ACP β-catenin-accumulating clusters with the enamel knot and the palisading epithelium with the inner enamel epithelium.

### Cell clusters orchestrate paracrine signalling within human and murine ACP

The molecular similarities between human clusters and the enamel knot prompted us to explore further whether the clusters may also act as signalling centres in human ACP tumours. In agreement with this notion, our RNA profiling revealed that human ACP clusters expressed high levels of members of the FGF, BMP and WNT families of secreted factors compared with either palisading epithelium or reactive glia [Suppl. Fig. 7 (Online Resource 3); Suppl. Table 2d, e (Online Resource 4)]. *SHH* was also highly expressed by the cluster cells (Suppl. Fig. 7a), as previously shown [1]. The activation of the WNT pathway in human ACP has been well documented, and we identified the expression of several WNT ligands (e.g. *WNT4*, *WNT5A*, *WNT6*, *WNT7A*, *WNT10A*, *WNT10B* and *WNT16*) in the clusters relative to the palisading epithelium or glial reactive tissue [Suppl. Fig. 7b (Online Resource 3); Suppl. Table 2d, e (Online Resource 4); adjusted *p* value ≤ 0.03]. To reveal the cells responding to FGF, TGFβ and BMP factors, we analysed the expression of the ligands and their receptors in our human RNA-Seq datasets and performed immunofluorescence against downstream signalling effectors indicating pathway activation.

**Fig. 4:**
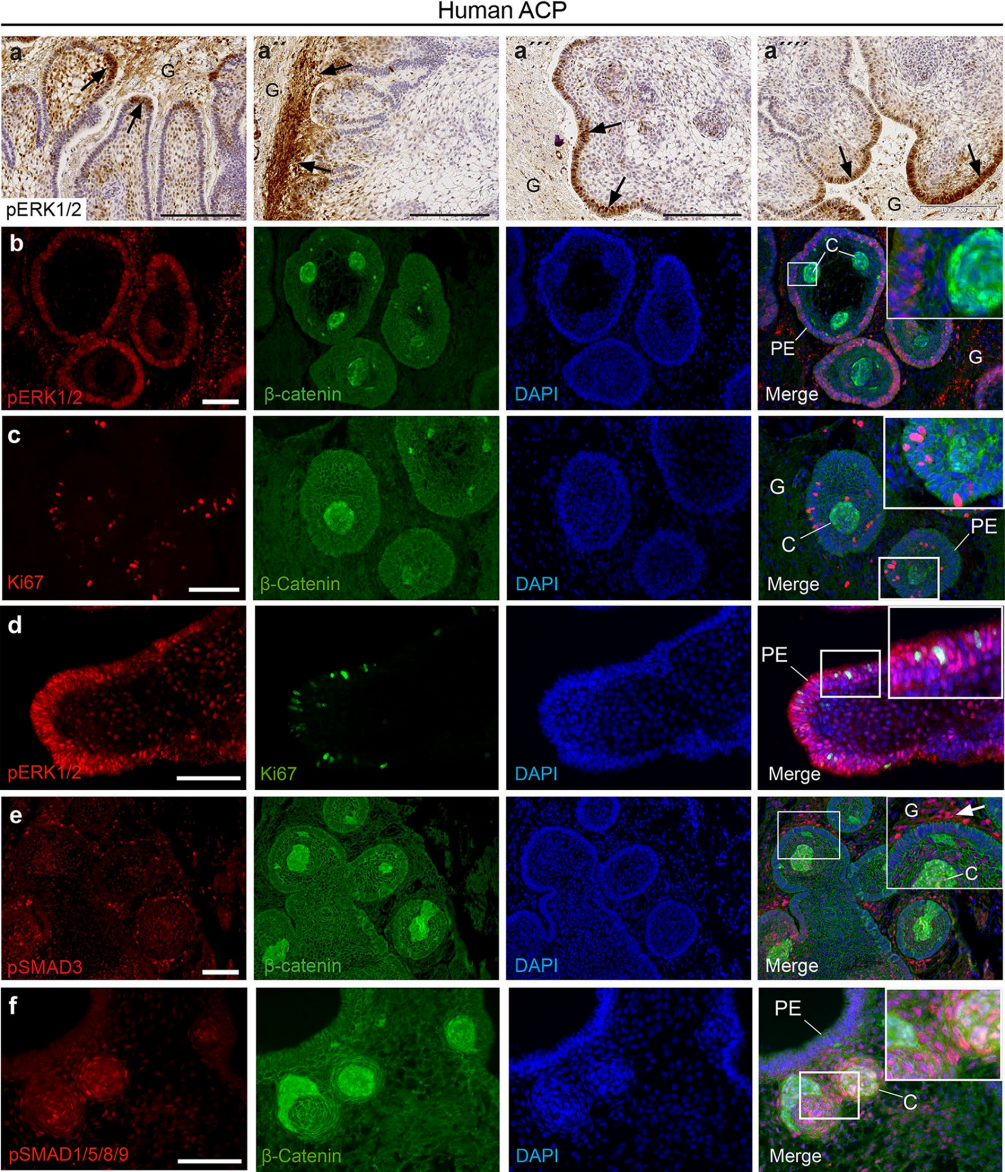
Identification of the activation of the MAPK/ERK, TGFB and BMP signalling pathways in human ACP. **a** Immunohistochemistry revealing the expression of phosphorylated ERK1/2 (pERK1/2), a read out of active MAPK/ERK pathway, at the tips of the invading tumour epithelium (palisading epithelium, arrows in **a**, **a**‴ and **a**″″) and within reactive glial tissue (G; arrows in **a**″). **b** Double immunofluorescent staining showing pERK1/2 expression in the palisading epithelium (PE) around the β-catenin accumulating clusters (C), which express several activating ligands of the MAPK/ERK pathway (see main text for details). Note that cells within the reactive glial tissue (G) are also pERK1/2 positive. **c** Double immunofluorescence revealing abundant Ki67+ve cells in the palisading epithelium close to clusters. **d** Double immunofluorescence showing Ki67 and pERK1/2 co-expression within the palisading epithelium (PE). **e** Double immunofluorescence showing pSMAD3 staining, indicating activation of TGFβ signalling, in both tumour and reactive glia, with strongest signal in reactive tissue adjacent to tumour epithelia (arrowhead). Double immunofluorescence reveals pSMAD1/5/9 staining, indicating BMP signalling in cells within and adjacent to the β-catenin-accumulating clusters (C). Note the absence of staining in the palisading epithelium (PE). Scale bars: **a**–**a**″″ 200 μm; **b**–**f** 100 μm

*FGFR1*–*3* were broadly expressed across tumour and glial cells and not differentially expressed between compartments [Suppl. Fig. 7b (Online Resource 3); Suppl. Table 2d, e (Online Resource 4)]. The ligands *FGF3*, *FGF4*, *FGF9*, *FGF12*, *FGF13*, *FGF18* and *FGF19* were highly expressed in the clusters relative to the palisading epithelium or the glial reactive tissue [adjusted *p* value ≤ 0.1; Suppl. Fig. 7a, b (Online Resource 3); Suppl. Table 2d, e (Online Resource 4)]. Downstream activation of the MAPK/ERK pathway, as evidenced by phosphorylation of ERK1/2, was identified by immunohistochemistry and immunofluorescence within the palisading epithelium around the clusters and neighbouring reactive tissue, but no positive signal was observed in the clusters themselves (Fig. 4a, b, d). Particularly prominent staining was detected at the leading edge of tissue invasion (Fig. 4a). Areas of palisading epithelium around the clusters were often enriched for the proliferation marker Ki67 (Fig. 4c), concurrent with elevated pERK1/2+ve staining (Fig. 4b). Double immunofluorescence of three human ACP samples revealed a variable degree of co-localisation between Ki67 and pERK1/2 expression (Fig. 4d) (tumour 1: 80%, 45 co-expressing cells out of 56 Ki67+ve cells; tumour 2: 44%, 26 co-expressing/59 Ki67+ve cells; tumour 3: 15%, 5 co-expressing/33 Ki67+ve cells).

*BMP4* and *BMP8A* were highly expressed by clusters (adjusted *p* value ≤ 0.001) and *BMPR1A* and *BMPR2* were expressed across all cell types, whist *BMPR1B* was highly expressed in the glial reactive tissue (adjusted *p* value = 0.00011) [Suppl. Fig. 7b (Online Resource 3); Suppl. Table 2d, e (Online Resource 4)]. Activation of the pathway, as evidenced by immunofluorescence staining against phosphorylated SMAD1/5/9 was widely present in tumour cells, particularly in proximity to the clusters, but was absent from the palisading epithelium (Fig. 4f). Other members of the TGF superfamily of secreted factors, such as *TGFB1*-*3*, as well as their receptors were broadly expressed across all tumour compartments, although *TGFB1* appeared non-significantly elevated in clusters relative to both PE (4.9-fold, unadjusted *p* value = 0.08; adjusted* p* value not assessed because of inadequate read numbers) and glial tissue (9.5-fold, adjusted *p* value = 0.37) [Suppl. Fig. 7a, b (Online Resource 3); Suppl. Table 2d, e (Online Resource 4)]. Double immunofluorescence against β-catenin and phospho-SMAD3 revealed the activation of the TGFβ pathway in tumour cells and reactive tissue. Increased signal was apparent within areas of reactive tissue closest to tumour cells (Fig. 4e).

**Fig. 5:**
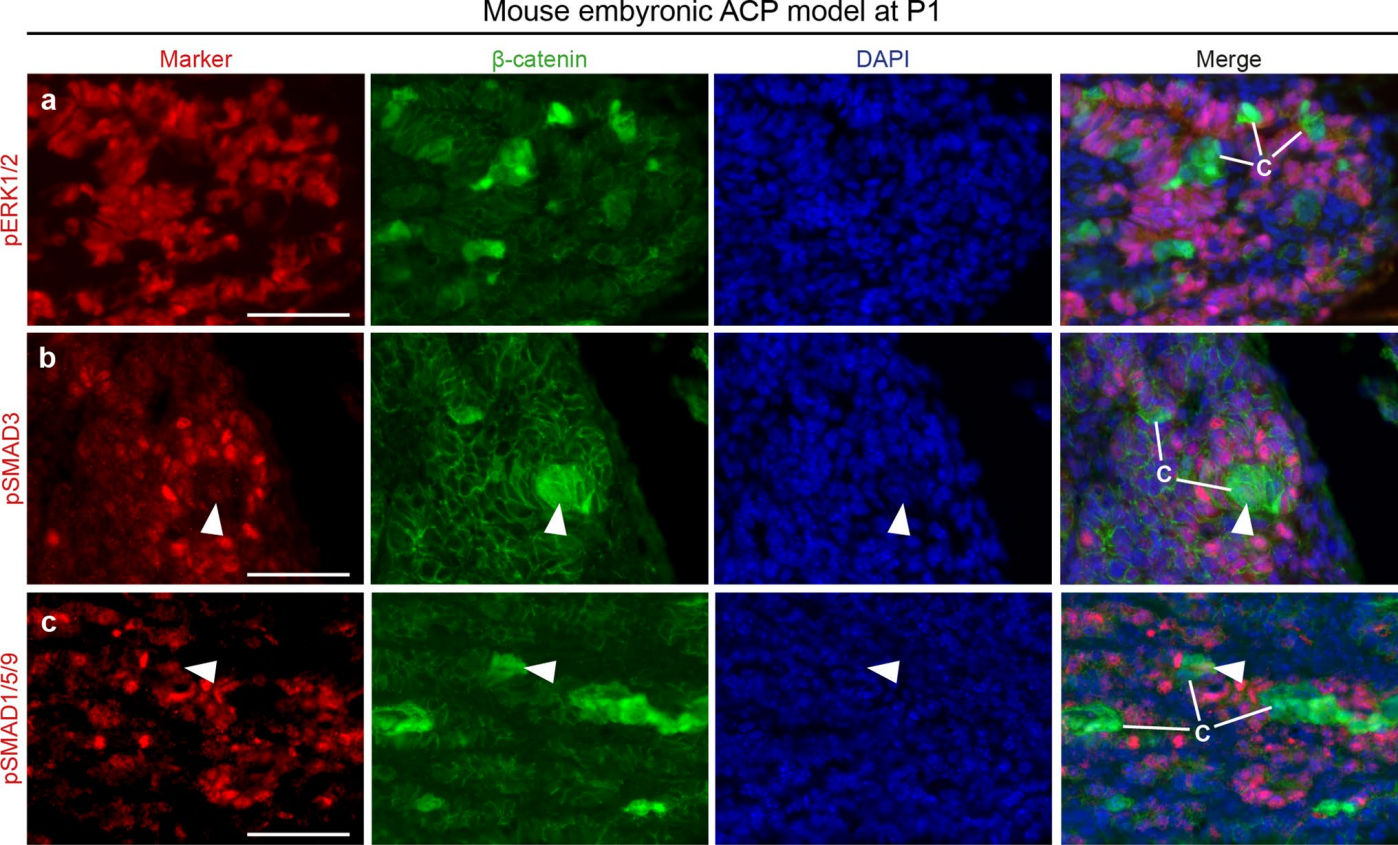
Identification of the activation of the MAPK/ERK, TGFB and BMP signalling pathways in the ACP embryonic mouse model. Double immunofluorescent staining on histological sections of neoplastic pituitaries of the ACP embryonic mouse model at postnatal day 1 (P1). Note the widespread expression of pERK1/2 in cells around the β-catenin-accumulating cell clusters, which show no expression of this marker. pSMAD3 and pSMAD1/5/9 staining is also predominant in cells surrounding the clusters, but occasionally weak staining is observed in some cluster cells (arrowheads). Scale bars 50 μm

We have previously shown that murine clusters in the *Hesx1*^*Cre/*+^*/Ctnnb1*^*lox(ex3)/*+^ embryonic model of ACP also express several genes of the FGF, TGFβ and BMP families [1], but the activation of these pathways has not been demonstrated so far. Immunofluorescent staining revealed strong expression of p-ERK1/2 in cells directly surrounding the β-catenin-accumulating clusters, which displayed no positive staining themselves (Fig. 5a). p-SMAD3 and p-SMAD1/5/9 staining was mostly observed in cells near the clusters, with some cluster cells showing weak staining (Fig. 5b, c). These analyses are consistent with the hypothesis that human and murine clusters act as signalling centres through the expression of multiple ligands that activate the SHH, WNT, MAPK/ERK, TGFβ and BMP signalling pathways in neighbouring cells.

### Preclinical studies provide support for an important role of the MAPK/ERK pathway in the pathogenesis of both human and mouse ACP

To further understand the role of the MAPK/ERK pathway in human ACP and to test its potential as a therapeutic target, we targeted this pathway using trametinib, a specific MEK inhibitor that has shown promising results in MAPK/ERK-driven solid tumours [34, 48] and currently being trialled in children and adolescents with a variety of solid tumours (NIH-NCI clinical trial: NCT02124772).

**Fig. 6:**
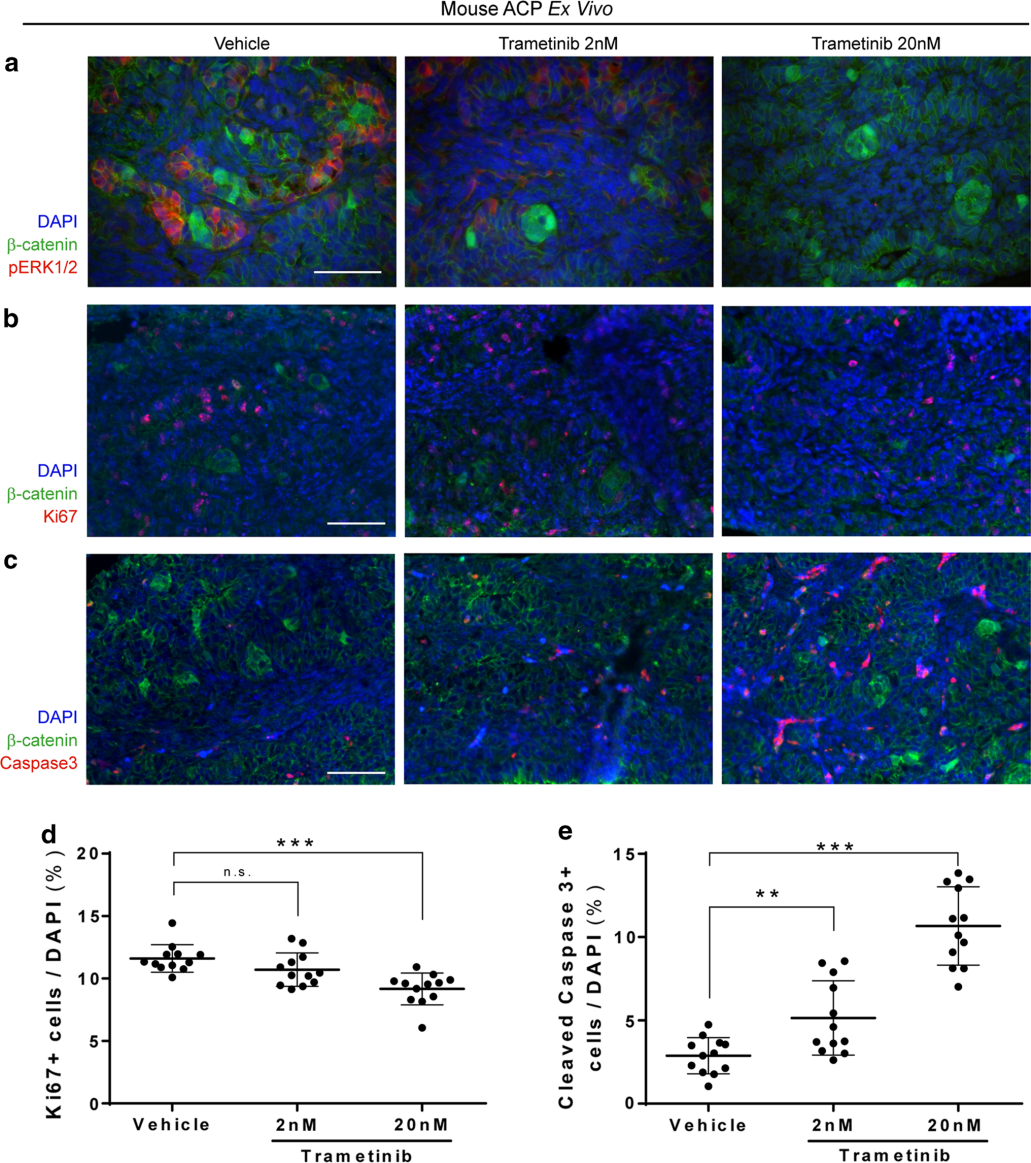
Ex vivo inhibition of the MAPK/ERK pathway in mouse ACP results in decreased proliferation and increased apoptosis of tumour cells. Neoplastic pituitaries of the ACP embryonic mouse model were cultured in the presence of the MEK inhibitor trametinib (2 or 20 nM) or the vehicle control (DMSO) for 18 h. Following histological processing, sections were immunostained against β-catenin and pERK1/2 (readout of active MAPK/ERK pathway; **a**), Ki-67 (proliferation marker; **b**) and cleaved caspase-3 (apoptosis marker; **c**). Quantitative analysis showing a significant dose-dependent reduction in Ki-67 proliferative index (**d**; 20 nM) and an increase in apoptosis (**e**; 2 and 20 nM) in trametinib-treated relative to vehicle-treated control. Kruskal–Wallis with Dunn’s post-test **p < 0.01; ***p < 0.001. Mean of 4.1 × 10^3^ nuclei for each point. Scale bars 50 μm

The neoplastic pituitaries of the *Hesx1*^*Cre/*+^*/Ctnnb1*^*lox(ex3)/*+^ mice at postnatal day 1 (P1) contain well-defined β-catenin-accumulating clusters. To test the effects of trametinib in the mouse model, we cultured neoplastic pituitaries at P1 in the presence or absence of 2 and 20 nM trametinib for 18 h (*n* = 6 pituitaries). P1 neoplastic pituitaries resemble human ACP more closely than older stages of murine tumour development at both the histological and molecular levels [4, 17, 40]. Immunofluorescence revealed an obvious decrease in p-ERK1/2 immunofluorescence within the neoplastic pituitaries upon trametinib treatment with both concentrations, which was more apparent at 20 nM, demonstrating the successful inhibition of the MAPK/ERK pathway (Fig. 6a). Assessment of proliferation revealed a significant reduction in the Ki67 proliferation index between the 20 nM trametinib-treated and vehicle control groups (vehicle, 11.6% ± 1.10; 2 nM trametinib, 10.7% ± 1.34; 20 nM trametinib, 9.17% ± 1.27; mean ± standard deviation; Kruskal–Wallis test followed by Dunn’s multiple comparison test; vehicle vs 2 nM *p* = 0.2438; vehicle vs 20 nM *p* = 0.00018) (Fig. 6b). Likewise, both trametinib treatments resulted in a dose-dependent significant increase in active caspase-3 immunofluorescence, a marker of apoptosis (vehicle, 2.88% ± 1.08; 2 nM trametinib, 5.15% ± 2.23; 20 nM trametinib, 10.7% ± 2.35; mean ± standard deviation; Kruskal–Wallis test followed by Dunn’s multiple comparison test; vehicle vs 2 nM *p* = 0.00756; vehicle vs 20 nM *p* = 0.000002) (Fig. 6c).

**Fig. 7:**
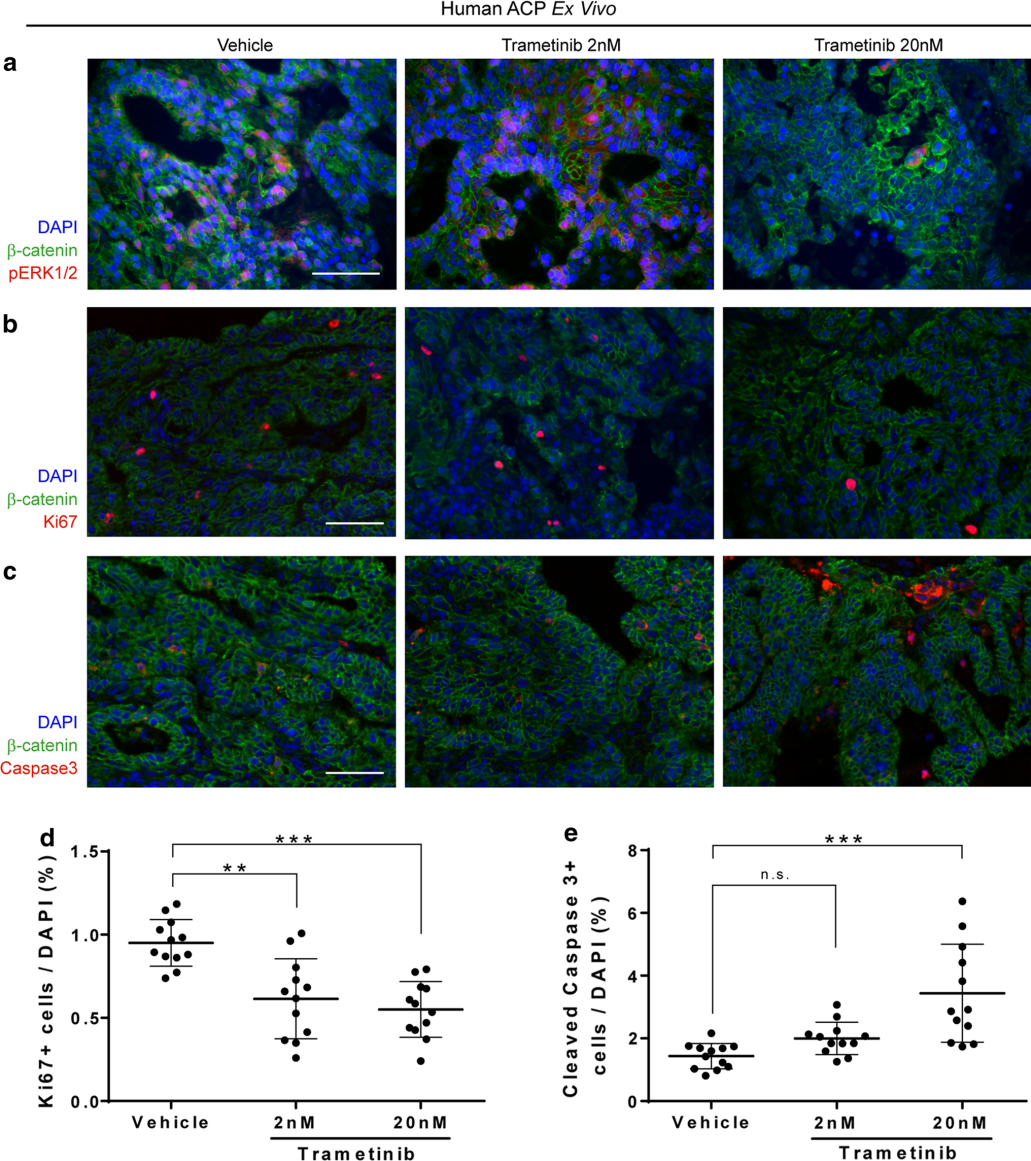
Ex vivo inhibition of the MAPK/ERK pathway in human ACP results in decreased proliferation and increased apoptosis of tumour cells. Small pieces of three human ACP tumours were cultured in the presence of the MEK inhibitor trametinib (2 or 20 nM) or the vehicle control (DMSO) for 18 h. Following histological processing, sections were immunostained against β-catenin and pERK1/2 (readout of active MAPK/ERK pathway; **a**), Ki-67 (proliferation marker; **b**) and cleaved caspase-3 (apoptosis marker; **c**). Quantitative analysis showing a significant dose-dependent reduction in Ki-67 proliferative index (**d**; 2 and 20 nM) and an increase in apoptosis (**e**; 20 nM) in trametinib-treated relative to vehicle-treated control. Kruskal–Wallis with Dunn’s post-test **p < 0.01; ***p < 0.001. Mean of 1.6 × 10^4^ nuclei for each point. Scale bars 50 μm

Next, we performed similar ex vivo culture experiments using small pieces of human ACP tumours, which were cultured with and without trametinib (*n* = 3 ACP tumours; 2 and 20 nM trametinib or vehicle control) for 18 h. Inhibition of the MAPK/ERK pathway was revealed by a reduction of p-ERK1/2 immunofluorescence in trametinib-treated tumours relative to vehicle-treated controls, which was more accentuated at 20 nM (Fig. 7a). Immunohistochemistry revealed a dose-dependent, significant decrease in the Ki67 proliferation index upon treatment with trametinib (vehicle, 0.950% ± 0.140; 2 nM trametinib, 0.614% ± 0.240; 20 nM trametinib, 0.551% ± 0.168; mean ± standard deviation; Kruskal–Wallis test followed by Dunn’s multiple comparison test; vehicle vs 2 nM *p* = 0.0028; vehicle vs 20 nM *p* = 0.00030). Similarly, assessment of apoptosis uncovered a dose-dependent increase in apoptosis, which reached significance at 20 nM (vehicle, 1.43% ± 0.404; 2 nM trametinib, 1.99% ± 0.514; 20 nM trametinib, 3.44% ± 1.56; mean ± standard deviation; Kruskal–Wallis test followed by Dunn’s multiple comparison test; vehicle vs 2 nM *p* = 0.07033; vehicle vs 20 nM *p* = 0.000052). Combined together, these preclinical studies provide preliminary evidence for a potential anti-tumoural effect of trametinib on ACP. Specifically, we show that trametinib treatment significantly reduces proliferation and increases apoptosis in both human and mouse ACP in vitro.

### Cytokine profiling of tumour and cystic fluid suggests activation of the inflammasomes in human ACP

**Fig. 8:**
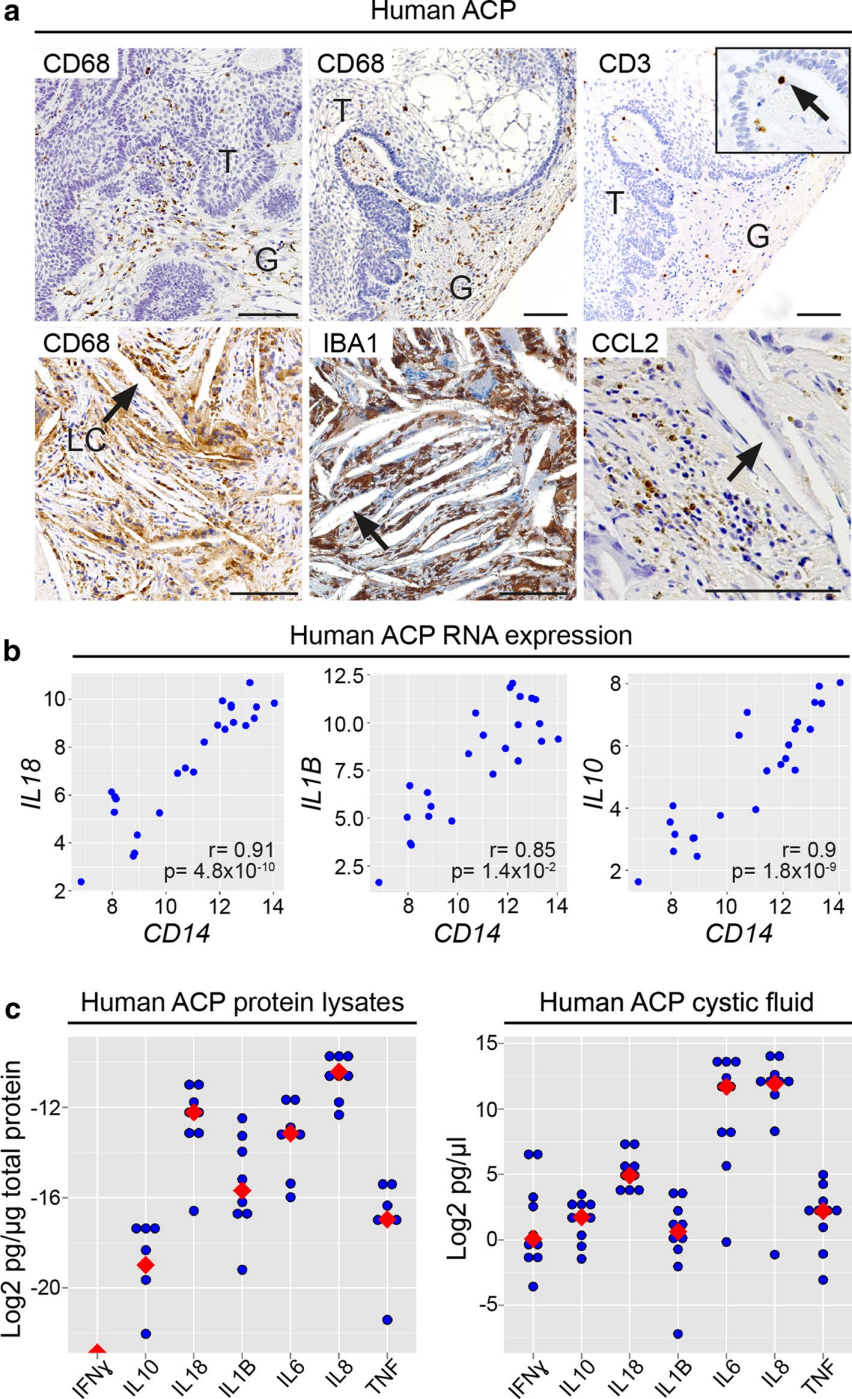
Characterisation of the immune microenvironment in the solid component and cystic fluid of human ACP. **a** Immunohistochemistry showing infiltration of myeloid (CD68+ve) and lymphoid (CD3+ve) within human ACP tumour (T) and reactive glial tissue (G). CD68+ve and IBA1+ve immunostaining is observed in close association with the cholesterol clefts (arrows). Likewise, immunohistochemistry against the chemokine CCL2 is detected near the cholesterol clefts. **b** The expression of the cytokines *IL18*, *IL1B* and *IL10* correlate significantly with *CD14* expression, a marker preferentially expressed in monocytes/macrophages, in the 24 samples (ACP tumours and control tissues) profiled by RNA-Seq. **c** Multiplex ELISA quantification of cytokine protein expression within the solid tumour (*n* = 8 tumours; left) and cystic fluid (*n* = 10 samples; right). IL8, IL18, IL6 and IL1B are the highest expressed cytokines in the solid tumour. In the cystic fluid, levels of IL6 and IL8 are the highest, but all the other cytokines are also detected. For solid tumour values were normalised against total protein and for cystic fluid samples against volume. The blue dots represent the value obtained of each cytokine for each sample and the red dots represent the median. Scale bars 100 μm

Our transcriptional analysis of whole ACP tumours exposed another major pattern of gene expression, the magenta module, which correlated with immune cell markers and was enriched for immune system genes [Fig. 1f; Suppl. Table 4a, k (Online Resource 6); Suppl. Fig. 2d (Online Resource 3)]. Supporting this finding, immunohistochemistry against CD68 and CD3 revealed myeloid-derived (CD68+ve) and lymphoid-derived (CD3+ve) cells variably infiltrating the reactive glial and tumour epithelial compartments within human ACP (Fig. 8a). Of note, myeloid cells, as evidenced by immunohistochemistry for CD68 or IBA1, were frequently observed in close association with the cholesterol clefts (Fig. 8a).

In addition, cytokine-encoding genes were highly up-regulated in ACP whole tumours compared with controls [Suppl. Table 6 (Online Resource 8)]. The expression of *IL1B*, *IL18* and *IL10* correlated significantly with the immune infiltrate and inflammatory cell markers, particularly CD14 and CD68 (*r* > 0.73, *p* < 1 × 10^−5^), as opposed to *CTNNB1* mutation allele frequency [Fig. 8b; Suppl. Table 6 (Online Resource 8)]. This suggests that cytokine expression predominantly derived from immune rather than tumour cells. The presence of cytokines in the ACP tumours was also assessed by multiplex ELISA against IL1B, IL6, IL8 (CXCL8), IL10, IL18, TNF (TNFα) and IFNG (IFNγ), which revealed the expression of all of these but IFNG in protein lysates from eight human ACPs (Fig. 8c). This is in line with recent findings [13]. Immunohistochemistry against IL1B, IL6 and IL8 did not yield reliable results in our hands using different antibodies.

The cystic fluid of ACP has been shown to contain inflammatory modulators [13, 59]. To explore the protein composition in greater detail, comprehensive proteome analysis was completed on cystic fluid from six ACP patients [Suppl. Materials and Methods (Online Resource 1)]. To obtain greater depth in the proteome coverage, samples of fluid were first subjected to a combinatorial peptide bead equalisation [Suppl. Fig. 8a (Online Resource 3)]. In total, 461 proteins were identified in all samples, using an FDR of 1% and requiring at least two unique peptides for each protein [Suppl. Table 7 (Online Resource 9)]. The overall proteome profile of all six cystic fluid samples was similar [Suppl. Fig. 8b (Online Resource 3)], covering an excess of five orders of magnitude. The most abundant proteins included albumin and several inflammation-associated proteins such as apolipoproteins, particularly APOA1 and APOA2, complement system proteins and immunoglobulins [Suppl. Fig. 8c (Online Resource 3]. Ontology analysis revealed an enrichment for terms related to immune/defence response, inflammation and sterol metabolism [Suppl. Fig. 8d (Online Resource 3)]. Cytokines were not detected in the proteome analysis, almost certainly attributable to their very low concentrations. We used multiplex ELISA to demonstrate the presence of IL1B, IL6, IL8, IL10, IL18, TNF and IFNG in the cystic fluid and whole ACP tumour protein lysates (Fig. 8c).

**Fig. 9:**
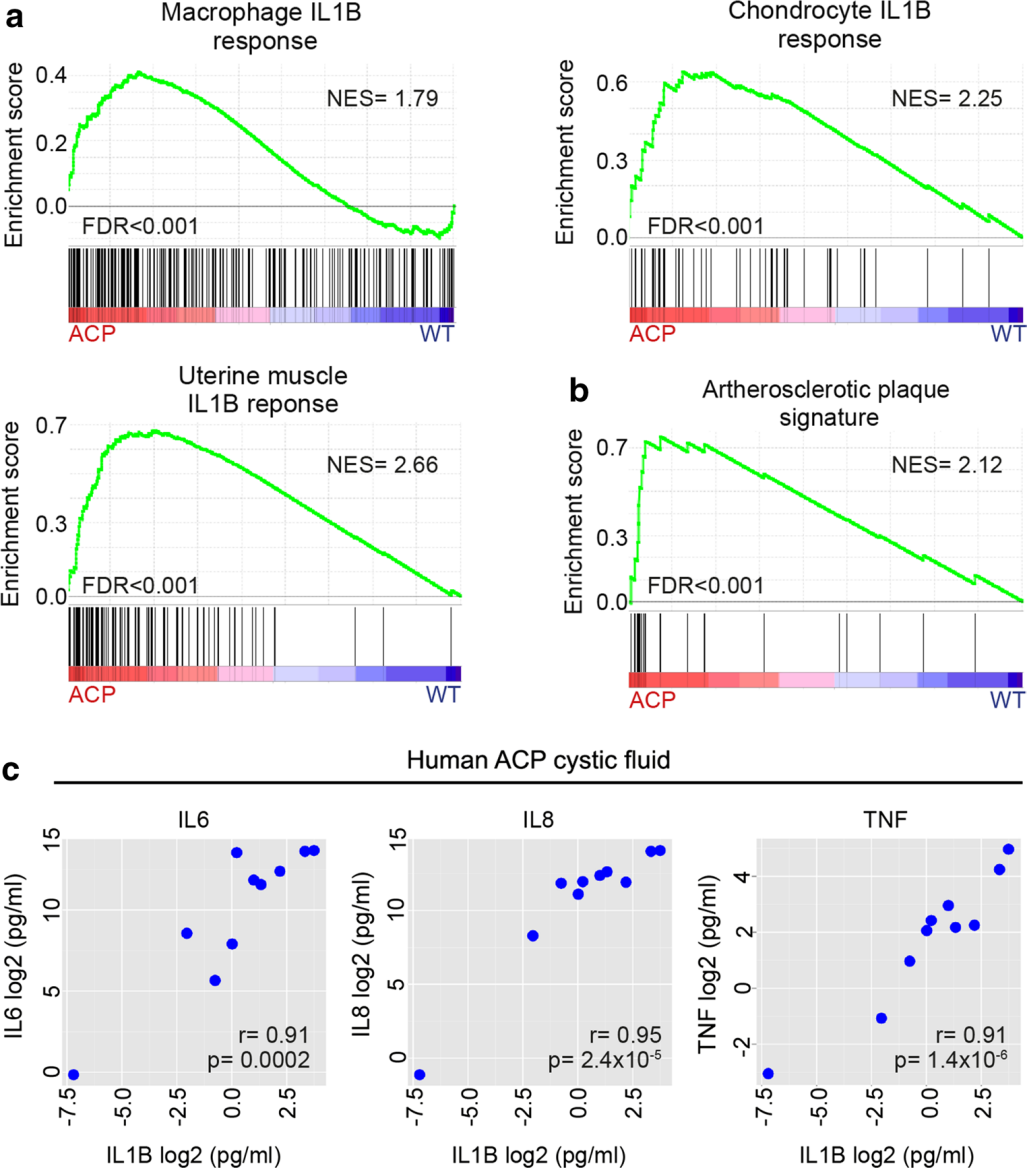
Activation of the inflammasomes underlies the ACP inflammatory response. **a** Gene set enrichment plots showing that human ACP tumours are enriched for genes expressed by macrophages, chondrocytes and uterine muscle cells exposed to IL1B in culture conditions. **b** Gene set enrichment plots reveal a molecular signature of atherosclerotic plaques in human ACP. Results are based on data obtained from RNA-Seq profiles from whole ACP tumours and control tissues (fetal pituitaries and NFPA). **c** The levels of IL6, IL8 and TNFα protein correlate with levels of IL1B in human ACP cystic fluid (*n* = 10 cystic fluid samples; determined by ELISA). NES normalised enrichment score, FDR false discovery rate

The cytokine profile identified in the ACP RNA-Seq dataset, in particular the significantly higher expression of *IL1A* (18.1-fold), *IL18* (14.8-fold), *TNF* (10.4-fold) and *IL1B* (7-fold) in human ACP tumours relative to controls, was suggestive of inflammasome activation [Suppl. Table 6 (Online Resource 8)]. Inflammasomes are innate danger-associated pattern recognition protein complexes that activate and up-regulate IL1 family members, particularly IL1B and IL18. This subsequently drives a pro-inflammatory response through secondary expression of pro-inflammatory cytokines including IL6, IL8, TNF and chemokines (e.g. CCL2) [5, 22, 37]. Several genes encoding core inflammasome components were all significantly expressed at higher levels in ACP tumours compared with control tissues, including *NLRP1* (6.4-fold), *NLRP3* (4.8-fold), *NLRC4* (4.8-fold), *CASP1* (5-fold) and *PYCARD* (4-fold) [Suppl. Table 2a (Online Resource 4)]. Moreover, GSEA highlighted a significant enrichment of the IL1B response signature in ACP tumours using three independent datasets of genes up-regulated upon IL1B treatment (NES = 1.79, 2.25, 2.66, respectively; FDRs < 0.001) (Fig. 9a). Complementing the mRNA expression data, protein levels of the downstream cytokines IL6, IL8 and TNF correlated with the protein levels of IL1B in ACP cystic fluid, supporting a role of IL1B in activating the inflammasomes (IL6, *r* = 0.91, *p* = 0.0002; IL8, *r* = 0.95, *p* = 2.4 × 10^−5^; TNFα, *r* = 0.96, *p* = 1.41 × 10^−5^) (Fig. 9c).

Having shown an expression profile, at both the mRNA and protein levels, supportive of the activation of inflammasomes in human ACP, we sought to explore the similarities of this molecular signature with that caused by other inflammasome activators. Cholesterol and other crystals (e.g. uric acid crystals) are established activators of inflammasomes, which mediate inflammation and are implicated in the pathogenesis of atherosclerosis and arthropathies (e.g. gout) [22, 33]. The ACP inflammatory cytokine profile was similar to that seen in gout, specifically regarding the high levels of IL1B, IL6 and IL18 [12]. Interestingly, we observed strong expression of the inflammasome-induced chemokine CCL2 in association with cholesterol clefts (Fig. 8a), while GSEA revealed a significant enrichment of an atherosclerotic plaque gene signature in ACP tumours relative to control tissues (NES = 2.12, FDR < 0.001) (Fig. 9b). Together, these results suggest an activation of the inflammasomes in human ACP and identify cholesterol as a possible activator.

## Discussion

**Fig. 10:**
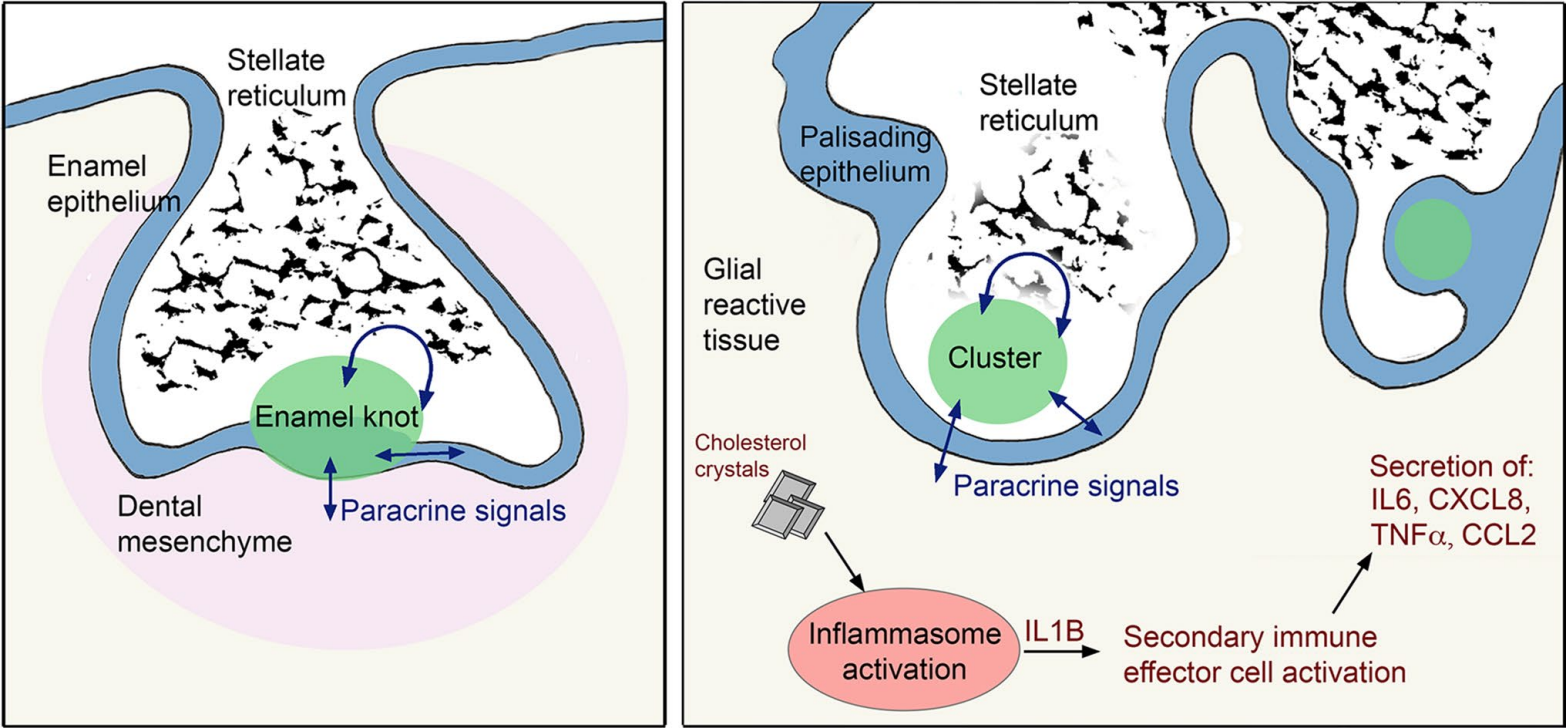
Schematic summary of the findings. Molecular and histological relationships between ACP pathogenesis and tooth development. The enamel knot and the β-catenin-accumulating clusters, which both have similar expression profiles and comparable histology, act as signalling hubs through the secretion of a several growth factors acting in an autocrine and/or paracrine manner on the surrounding cells, i.e. the enamel epithelium/dental mesenchyme in the forming tooth or the palisading epithelium, stellate reticulum and glial reactive tissue in ACP. Reciprocal signalling from surrounding tissues to the enamel knot and clusters is indicated by double-headed arrows. In the glial reactive tissue, cholesterol crystals activate the inflammasomes resulting in the secretion of IL1B, which in turn acts on the local immune effector cells to drive an inflammatory response

In this study, we have revealed the molecular signatures of different compartments in human ACP, including the β-catenin-accumulating cell clusters, palisading epithelium, glial tissue and the immune microenvironment. Through these analyses, we have identified and validated the expression of novel ACP genes, demonstrated the molecular similarities between human ACP and tooth development, revealed the MAPK/ERK pathway and inflammasome signalling as two novel targetable pathways and importantly, provided preliminary data supporting the use of MEK inhibitors against human ACP. A schematic summary of the findings of this research is shown in Fig. 10.

We provide a rationale that explains the long-standing observation of the histological similarities of ACP tumours with tooth development and odontogenic tumours. We show that ACP β-catenin-accumulating clusters and the enamel knots of developing teeth are molecularly analogous structures, as are ACP palisading epithelium and dental inner enamel epithelium. These pairs share a molecular signature and activate similar genetic programmes. Through the use of mouse models of ACP, we show that the expression of oncogenic β-catenin leading to the activation of the WNT pathway is sufficient to induce enamel epithelial/ameloblast-like gene expression and enamel knot-like cluster formation in Rathke’s pouch derivatives. Importantly, when the same degradation-resistant form of *Ctnnb1* (*Ctnnb1*^*lox(ex3)*^) is activated in the enamel epithelia under the control of the *Krt14* or *Sox2* promoters, the result is continuous tooth formation, with the presence of multiple enamel knots and with morphological similarities to ACP [27, 58]. Of note, fully formed teeth including dentin are occasionally seen in human ACP [6, 42, 49].

Analogous to the developing tooth, we highlight a complex system of paracrine signalling occurring between tissue compartments, centred on ACP clusters. We characterise the FGF, MAPK/ERK, TGFβ, BMP and EDAR signalling pathways in ACP. The importance for many of these genes and pathways has been functionally explored in the developing tooth, where their manipulation alters the number of teeth and/or their morphogenesis [20, 28, 38, 55, 57]. As the consequences of gene inactivation have been more extensively studied in the tooth development field, it is plausible to speculate that this knowledge may inform on the genes/pathways that may be more relevant in the pathogenesis of human ACP.

Illustrating this concept, we show that ex vivo MEK inhibition with trametinib results in decreased proliferation increased apoptosis in both mouse and human ACP. Likewise, individuals with Costello syndrome, a condition that results from germline activating mutations in *HRAS* leading to the over-activation of the MAPK/ERK pathway, show defective enamel mineralisation with increased number, proliferation and irregular orientation of ameloblasts [18]. This phenotype can be rescued by MEK inhibition in murine models of Costello syndrome [18]. We show that MAPK/ERK pathway inhibition ex vivo using trametinib is associated with decreased proliferation and increased apoptosis in both mouse and human ACP, suggesting that other pathways of importance during tooth development may also be relevant in the context of ACP pathogenesis. Future preclinical studies in both genetic and patient-derived xenograft mouse models of ACP will assess the effects of trametinib treatment in tumour development.

Finally, we highlight that inflammasome activation may underlie the inflammation observed in human ACP, and identify that cholesterol crystals may be a potential inflammasome activator (Fig. 10). This finding could have important therapeutic consequences. Prevention of inflammasome activation by the use of IL1R inhibitors (e.g. anakinra) has been remarkably effective in several autoinflammatory diseases, most notably in cryopyrin-associated periodic syndromes (CAPS), gout and atherosclerotic disease [15, 44, 47, 52]. Inhibitors such as anakinra are used in routine clinical practice, including in paediatrics, and are known to cross the blood brain barrier and improve neuroinflammation in patients with severe CAPS [15, 44]. The ability to measure downstream cytokines e.g. IL6 and IL8 within cystic fluid offers an opportunity to develop biomarkers of therapy response to anakinra and other similar drugs. In summary, we reveal a detailed molecular rationale underpinning the resemblance of ACP to tooth development and odontogenic tumours, and additionally, we provide evidence from preclinical testing of the use of novel targeted therapies in ACP, opening the door for biologically driven studies in human patients.

## Electronic supplementary material

Below is the link to the electronic supplementary material.

Supplementary material 1 (DOCX 89 kb)

Supplementary material 2 (DOCX 23 kb)

Supplementary material 3 (XLSX 11697 kb)

Supplementary material 4 (DOCX 134 kb)

Supplementary material 5 (XLSX 2200 kb)

Supplementary material 6 (DOCX 134 kb)

Supplementary material 7 (XLSX 15 kb)

Supplementary material 8 (XLSX 201 kb)

Supplementary material 9 (PDF 16461 kb)
