# Supplementary material for: Tumour compartment transcriptomics demonstrates the activation of inflammatory and odontogenic programmes in human adamantinomatous craniopharyngioma and identifies the MAPK/ERK pathway as a novel therapeutic target
Source: Acta Neuropathol. 2018 Mar 14;135(5):757–77. doi: 10.1007/s00401-018-1830-2 (PMC5904225; doi:10.1007/s00401-018-1830-2)
Supplement: Supplementary file 1 — Supplementary material 1 (DOCX 89 kb) [file 401_2018_1830_MOESM1_ESM.docx]

**Supplementary Text (Online Resource 1)**

**Tumour compartment transcriptomics demonstrate the activation of inflammatory and odontogenic programmes in human adamantinomatous craniopharyngioma and identify the MAPK/ERK pathway as novel therapeutic target**

 John R. Apps^1,2,*^, Gabriela Carreno^1^, Jose Mario Gonzalez-Meljem^1,3^, Scott Haston^1^, Romain Guiho^1^, Julie E. Cooper^1^, Saba Manshaei^1^, Nital Jani^4^, Annett Hölsken^5^, Benedetta Pettorini^6^, Robert J. Beynon^7^, Deborah M. Simpson^7^, Helen C. Fraser^1^, Ying Hong^8^, Shirleen Hallang^9^, Thomas J. Stone^1,2^, Alex Virasami^2^, Andrew M. Donson^10^, David Jones^11^, Kristian Aquilina^12^, Helen Spoudeas^13^, Abhijit R. Joshi^14^, Richard Grundy^15^, Lisa CD Storer^15^, Márta Korbonits^16^, David A. Hilton^17^, Kyoko Tossell^18^, Selvam Thavaraj^19^, Mark A. Ungless^18^, Jesus Gil^18^, Rolf Buslei^5,20^, Todd Hankinson^10^, Darren Hargrave^21^, Colin Goding^22^, Cynthia L. Andoniadou^23,24^, Paul Brogan^8,25^, Thomas S. Jacques^1,2^, Hywel J. Williams^4^ and Juan Pedro Martinez-Barbera^1,*^

1) Developmental Biology and Cancer Programme, Birth Defects Research Centre, UCL Great Ormond Street Institute of Child Health, University College London, London, UK

2) Histopathology Department, Great Ormond Street Hospital NHS Trust, London, UK

3) Basic Research Department, National Institute of Geriatrics, Mexico City, Mexico

4) Centre for Translational Omics - GOSgene, Genetics and Genomic Medicine Programme, UCL Institute of Child Health, University College London, London, UK

5) Department of Neuropathology, Friedrich-Alexander University Erlangen-Nürnberg (FAU), Erlangen, Germany

6) Alder Hey Children’s Hospital NHS Foundation Trust, Liverpool, UK

7) Centre for Proteome Research, Institute of Integrative Biology, University of Liverpool, UK

8) Infection, Immunity and Inflammation Programme, UCL Great Ormond Street Institute of Child Health, University College London, London, UK

9) Centre for Craniofacial and Regenerative Biology, King's College London, London, UK

10) Department of Pediatrics, University of Colorado Anschutz Medical Campus, Aurora, Colorado

11) German Cancer Research Center (DKFZ), Heidelberg, Germany

12) Neurosurgery Department, Great Ormond Street Hospital NHS Trust, London, UK

13) Endocrinology Department, Great Ormond Street Hospital NHS Trust, London, UK

14) Laboratory Medicine, Royal Victoria Infirmary, Newcastle, UK

15) Children’s Brain Tumour Research Centre, University of Nottingham, Nottingham, UK

16) William Harvey research Institute, Barts and the London School of Medicine and Dentistry, Queen Mary University, London, UK

17) Pathology Department, Plymouth Hospitals NHS Trust, Plymouth, UK

18) MRC London Institute of Medical Sciences, Imperial College London, London, UK

19) Head and Neck Pathology, Dental Institute, King's College London, London, UK

20) Institute of Pathology, Klinikum Sozialstiftung Bamberg, Bamberg, Germany

21) Haematology and Oncology department, Great Ormond Street Hospital NHS Trust, London, UK

22) Ludwig Institute for Cancer Research, Oxford University, Old Road Campus, Headington, Oxford, UK

23) Centre for Craniofacial and Regenerative Biology, King’s College London, Guy’s Hospital, Floor 27 Tower Wing, London, UK

24) Department of Internal Medicine III, Technische Universität Dresden, Fetscherstaße 74, 01307 Dresden, Germany

25) Rheumatology department, Great Ormond Street Hospital NHS Trust, London, UK

* Corresponding authors: John Apps [j.apps@ucl.ac.uk](mailto:j.apps@ucl.ac.uk), JP Martinez-Barbera, [j.martinez-barbera@ucl.ac.uk](mailto:j.martinez-barbera@ucl.ac.uk). +44 (0) 207 905 2821

**SUPPLEMENTARY RESULTS**

**Gene expression modules**

The expression patterns of each module were plotted as heatmaps affirming the patterns highlighted in **Fig. 1f** (**Suppl. Figs. 2-4 (Online Resource 3)**). Full details of genes within each module as well as the enriched ontologies can be found in **Suppl. Table 4 (Online Resource 6)**. The brown and magenta modules are discussed in further detail in the main text. Below we describe the remaining modules, including further analysis of the blue and dark-turquoise modules.

**Black and dark grey modules**

WGCNA analysis showed that the black module also correlated with tumour content (r=0.65, p=6x10^-4^), similar to the brown module but at a lesser extent. In addition, the black module correlated better with abundance of wet keratin (r=0.73, p=1x10^-5^) (**Fig. 1f; Suppl. Fig. 2a,b (Online Resource 3)**). The heatmap showed the highest expression of the black module genes predominantly in those tumour cases where wet keratin had been identified as a predominant feature (**Suppl. Fig. 2b (Online Resource 3)**). This module includes several keratins, keratin associated proteins and enamel proteins and proteinases (**Suppl. Table 4a,d (Online Resource 6)**). Likewise, the dark grey module also correlated with *CTNNB1* mutation allelic frequency (r=0.78, p=6x10^-6^), but some genes were also expressed in control tissues (**Suppl. Fig. 2c (Online Resource 3)**). This module includes genes involved in cell-cell junction organisation and skin development (**Supplementary Table 4a,f (Online Resource 6)**). Among the brown, black and dark grey module, the brown module is more likely to specifically represent the molecular signature of the tumour cell compartment, hence excluding host-derived reactive tissue. We explore this module further in the main text. Black module genes are also further explored with respect to odontogenesis.

**Blue and dark turquoise modules**

Both the blue and dark turquoise modules, which closely correlated with the presence of glial tissue through WGCNA, were enriched for genes implicated in central nervous system development (**Fig. 2f; Suppl. Table 4a,j,l (Online Resource 6)**). This suggests that these genes are expressed by the reactive glial component of the specimens. This was validated by GSEA, which showed a significant enrichment of expression of the genes in these modules in laser capture micro-dissected reactive glial tissue when compared with isolated tumour tissue (i.e. clusters and palisading epithelium together) (NES: Blue=3.36, Dark Turquoise=3.07, FDR <0.001) (**Fig. 2d**). Blue module genes were only highly expressed in tumours with glial tissue (**Suppl. Fig. 3a (Online Resource 3)**), whereas dark-turquoise genes were also highly expressed in fetal pituitaries (**Suppl. Fig. 3b (Online Resource 3)**). This suggests that the genes in these modules may indicate different biological functions. Genes with strong membership of the blue module included the astrocytic markers *S100B* and GFAP, whilst the gene with the highest module membership score for the dark turquoise module was *NKX2.2* a transcription factor involved in oligodendrocyte differentiation suggesting these modules represent different glial cell types.

**Green module**

Expression of the genes in this module correlated with *GFAP* expression (r=0.64, p=8x10^-4^) and inversely with tumour content (r=-0.7, p=2x10^-4^) and *CTNNB1* mutation frequency (r=-0.9, p=2x10^-9^ (**Fig. 1f**). The green module genes were highly expressed by tumours with abundant glial tissue and also by control tissues (**Suppl. Fig. 3c (Online Resource 3)**). It was enriched for genes of microtubules and cilia (**Supplementary Table 4a,m (Online Resource 6)**).

**Pink, cyan and light green modules**

Genes of the pink, cyan and light green modules correlated with fetal pituitary and/or NFPA and were expressed by the fetal pituitaries and/or NFPA control tissues (**Fig. 1f,** **Suppl. Fig. 4a-c (Online Resource 3)**). Genes expressed in these modules included genes related to hormone processing and release (pink, light green) or cell cycle (cyan), which are cellular processes relevant to these control tissues (i.e. containing hormone producing and proliferative cells (**Suppl. Table 4a,b,h,I (Online Resource 6)**).

**Yellow module**

Genes in this module correlated weakly with fetal pituitaries and inversely with NFPA and were expressed by fetal pituitaries and variably across ACPs (**Fig. 1f,** **Suppl. Fig. 3d (Online Resource 3)**). Genes in this module included collagen and extracellular matrix genes (**Suppl. Table 4a,c (Online Resource 6)**).

**Grey module**

The grey module contains the remaining 131 genes that could not be grouped into a distinct co-expression module. It included a small subset of genes relating to sex determination (e.g. *XIST*, *TSIX*, *PRKY*, *ZFY*), information regarding patient sex was only available in a small number of cases and where available was consistent with expression of these genes (**Suppl. Fig. 4d (Online Resource 3)**).

**Modular analysis of an independent ACP transcriptomics dataset**

WGCNA was also performed on the human ACP microarray data published by Gump *et al.* to assess whether the co-expression modules observed in our cohort were also present in this independent dataset [6]. Of the 5000 genes included in our WCGNA analysis, the expression patterns of 2963 were available but the other genes were not present in the microarray data provided [6]. Multidimensional scale plotting and hierarchical clustering of genes expression patterns showed consistency with the modules identified in our cohort (**Suppl. Fig. 1b (Online Resource 3)**). Moreover, statistical assessments of module preservation, which measure how well patterns of gene co-expression are maintained in independent sample sets, revealed a moderate preservation of the major modules between the two datasets (i.e. brown, magenta, blue; Zsummary ≥ 10) (**Suppl. Fig. 1c (Online Resource 3**)). Together, these computational analyses suggest that the molecular signatures of the main cellular types within ACP samples (i.e. tumour epithelium, reactive glial tissue and inflammatory infiltrate) may be contained in specific modules.

**SUPPLEMENTARY MATERIALS AND METHODS**

**Human tumour and pituitary samples**

Frozen samples were subdivided into two, one part for confirmation of diagnosis and estimation of the tumour content, and another for RNA extraction and sequencing. The percentage of tumour content (i.e. palisading epithelium, stellate reticulum and clusters) and non-tumour content (i.e. glial reaction and wet keratin), as assessed histologically for each of the tumour samples, ranged from 20% (e.g. JA011) to 90% (e.g. JA014). Eight samples contained abundant glial reactive tissue and wet keratin was variably present in the frozen sections and prominent in eight cases (**Suppl. Table 1 (Online Resource 2); Fig. 1a**). Tumour content was estimated as the proportion of tumour cell nuclei within the whole of the frozen section (i.e. including glial reactive tissue). Cell nuclei, rather than area were counted to compensate for the lower cell density of the host-derived glial reactive tissue relative to the tumour. RNA was extracted using the miRNeasy Mini kit (Qiagen). Experiments were performed under NHS Research Ethics Committee approval (14/LO/2265; 13/NW/0851) or approval from individual biobanks. Where required, informed consent was obtained from individual participants included in the study.

**Laser capture micro-dissection of human ACP**

Cryopreserved ACP 8-10μm histological sections were mounted on membrane slides (Zeiss Membrane Slides 1.0 PEN, Item No 415190-9041-00) and stained with haematoxylin and eosin. The Zeiss PALM MicroBeam system was used to separately isolate multiple epithelial whorls/clusters and areas of palisading epithelium, and neighbouring reactive glial tissue, all of which were collected on adhesive caps (Zeiss, Adhesive caps opaque 415190-9201-001). Blood vessels and areas of evident inflammation were avoided. RNA was extracted using the RNeasy Microkit (Qiagen). For case JA004 collection and processing of epithelial whorls/clusters and palisading epithelium was performed twice, independently, as a technical control for assessing the reproducibility of the RNA-Seq data when using very low amounts of total RNA.

**RNA-Sequencing and targeted DNA sequencing**

Sequencing libraries were prepared using the TruSeq Stranded mRNA Library Prep kit (Illumina) and sequenced to a depth of approximately 50 million paired-end reads of 80 base pairs. For laser capture micro-dissected samples RNA was amplified using the Clontech SMARTer® low input RNA kit and sequencing libraries generated using the Nextera XT library prep and sequenced to a depth of approximately 15 million 76 base pair single-end reads. For murine ACP samples, sequencing was performed by the Oxford Wellcome Trust Centre for Human Genetics. Sequencing libraries were prepared using the TruSeq Stranded mRNA library Prep kit (Illumina) and sequencing was performed to a depth of approximately 30 million paired-end reads of 76 base pairs. Targeted DNA sequencing was carried out as described [15]

**Bioinformatics analysis**

FASTQ files were assessed using FASTQC and aligned using STAR aligner against UCSC human reference genome hg19 or murine reference genome GRCm38.ERCC. Exon 3 *CTNNB1* mutations were assessed using the integrative genome viewer (Broad Institute) and the variant allele frequency calculated as a proportion of all reads of a given base. FASTQ and BAM files are available on Array Express (E-MTAB-5267 and E-MTAB-5266). Downstream analyses were performed using Bioconductor in RStudio. Full code is available on request.

**Differential expression analysis**

Differential expression analysis was performed between ACP tumours and control tissues. For control tissues, fetal pituitary was selected as the most likely to reflect the cell of origin of ACP tumours [2, 5]. Non-functioning pituitary adenoma (NFPA) was also selected as previous microarray studies have shown that it closely resembles adult pituitary [6]. Using Bioconductor, per gene read counts were calculated using RSubread, differential analyses performed using DESEq2, ontology analysis using GOSeq [12, 13, 20]. Differential expression was performed using a standard DESeq2 pipeline. This recommends use of adjusted p-value cut of p<0.1 (see Love et al., 2014 for details [13]). This adjusted p-value is calculated using the procedure of Benjamini and Hochberg following "independent filtering" of genes, which have inadequate reads to reach significance [13].  Ontology relationships were explored using Quick GO [1]. For gene set enrichment analysis (GSEA), genes were ranked by the Wald statistic and GSEA performed using the pre-ranked tool in GSEA v2.2 (Broad Institute) [16]. The WNT pathway and inflammatory repsonse gene sets were downloaded from the hallmark molecular signatures database v5.2 (Broad Institute).

Enamel epithelium ameloblast and enamel knot gene sets were derived from a gene expression tooth database [7, 16]. This curated gene set includes details of genes that have been experimentaly confirmed to be expressed in specific cell types at different developmental stages (e.g. by *in situ* hybridisation in various species, including human, mouse and vole). Where required gene names were converted to their human homologues. IL1β response gene sets were derived from published expression analyses, which assessed the response to IL1β in macrophages, myometrium or osteochondroma cells [3, 9, 18]. An atherosclerosis gene set was taken from expression array analysis identifying 29 genes up-regulated in atherosclerotic plaques (aorta, carotid or femoral) compared to non-atherosclerotic plaques (Fold change > 15, FDR<0.05) [17].

**Weighted gene co-expression network analysis (WGCNA)**

Initially, we performed WGCNA using variance stabilised transformed counts (a method of count normalisation in DESeq2) of all the genes in our RNA-Seq dataset or just the top 5000 most variably expressed genes as assessed by their median absolute deviation. As results were very similar, for computational ease, we decided to perform all the subsequent analyses with the of the top 5000 most dysregulated genes. WGCNA is a systems biology approach used to identify co-regulated gene networks across datasets in a hypothesis independent manner [8, 10, 21]. First described in the context of brain tumours, this approach has been used to identify novel candidate pathogenic genes [8, 10, 21]. Genes whose patterns of expression across samples are similar are grouped together in modules based on their Pearson correlation coefficient. WGCNA uses soft thresholding to weight/emphasize high correlations at the expense of low correlations, generating an adjacency measure for each gene. Groups of closely interconnected (co-expressed) genes, as assessed using a network analysis function (topographical overlap measure) are then defined as modules and assigned a colour. Full details are available in Langfelder and Horvath, 2008 [10].

For each module an eigengene was calculated as a representative number of the gene expression of the whole module. This was correlated with available sample phenotype information of the tumours (e.g. diagnosis, histological features, *CTNNB1* mutation allele frequency, age, RNA integrity number (RIN)). Ontology analysis of modules was performed using the GOStats package [4]. Central nervous system cell gene sets were extracted from Zhang *et al.* and the immune infiltrate of breast cancer gene set from Yoshihara *et al.* [19, 22]. Gene enrichment scores and barcode plots were generated using the geneSetTest and barcodeplot functions in Limma [14]. Genes were ranked by their module membership score of a specific module, a measure of the extent to which a given genes expression pattern matches that of the module.

**Analysis of ACP published datasets**

Raw CEL files of ACP and comparable controls (3 normal pituitaries, 3 pituitary adenomas) published by Gump *et al*. were downloaded from the National Centre for Biotechnology Information Gene Expression Omnibus (GEO) database (accession numbers GSE68015, GSE26966) [6]. Expression values were robust multi-array average (RMA) normalised and WGCNA performed as above. Hierarchical clustering and multidimensional scaling plots of the expression patterns of the genes were generated where genes were annotated using the module colours from the analysis of our RNA sequencing results. Module preservation analysis was performed as per Langfelder *et al,* 2011 [11]. The Zsummary statistic summarises several different statistical measures of how well preserved modules are in independent datasets.

**Proteomic analysis of cystic fluid**

The proteome of the cystic fluid is dominated by a few, highly abundant proteins that impede detection of lower abundance proteins. Accordingly, proteomic profiling of cystic fluid was based on proteome equalisation using Proteominer beads (Bio-Rad) comprising a large, highly diverse bead-based library of combinatorial peptide ligands that simultaneously suppress high abundance proteins and which concentrate low abundance proteins. Preliminary analyse of a sample without Proteominer bead equalisation allowed detection of 50 proteins, but after equalisation, this increased to approximately 500 proteins. A 20%(v/v) ethanol suspension of Proteominer beads was mixed on the Coulter mixer and 50µL was dispensed in to 1.5mL low bind tubes (equal to 10 µL resin). The resin was centrifuged at 1,000 x g for 1min and supernatant ethanol aspirated and discarded. The resin was then washed 3x with 100µL of PBS pH7.4. Cyst fluid (~110µL) were centrifuged at 10,000 x g for 10min to remove any particulates and 100µL of each sample added to ~10 µL resin in each tube and thoroughly mixed. The tubes were placed in a rotary mixer for 2h at RT. Samples were centrifuged at 1,000 x g for 1 min and the supernatant was aspirated and retained. The beads were washed 3x with 100 µL of phosphate buffered saline and then finally in 100 µL of 25 mM ammonium bicarbonate buffer (ambic). The beads were re-suspended in 80µL of 25 mM ambic and 5 µL of freshly reconstituted 1%(w/v) Rapigest SF (a digestion enhancer, Waters, UK) added and the sample was heated at 80°C for 10 min. For reduction, 5 µL of 60 mM DTT in 25 mM ambic was added and the sample incubated at 60°C for 10min, followed by addition of 5µL of 180 mM iodoacetamide in 25 mM ambic and incubation at room temperature in the dark for 30 min. Trypsin (5µL of a 0.2 mg/mL stock solution in 50 mM acetic acid) was added and the sample was incubated overnight. The digests were acidified with trifluoroacetic acid (final concentration 1 % (v/v)) and incubated for 45 min at 37°C before centrifugation at 17,200 x g for 30 min, after which the clarified digest was aspirated into a fresh 0.5 mL low-bind tube. After dilution of the digest 5-fold with, 1µL was analysed by LC-MS/MS. To provide an estimate of the amount of each protein in each sample, each digest was diluted with a protein standard digest (50 fmol/µL yeast enolase digest) in 0.1% (v/v) trifluoroacetic acid/ 3% (v/v) acetonitrile. However, these calculated values reflect the equalised loading onto the combinatorial library beads, not the initial concentrations in the cystic fluid.

The peptide mixture was resolved on an Ultimate 3000 nano system (Dionex/Thermo Fisher Scientific) connected to a QExactive mass spectrometer. For each analysis, the sample was loaded onto a trap column (Acclaim PepMap 100, 2 cm x 75 µm inner diameter, C_18_, 3 µm, 100Å) at 9µL/min with an aqueous solution containing 0.1%(v/v) TFA and 2%(v/v) acetonitrile. After 3 min, the trap column was set in-line with an analytical column (Easy-Spray PepMap® RSLC 50 cm x 75 µm inner diameter, C_18_, 2 µm, 100Å) (Dionex). Peptide elution was performed by applying a mixture of solvents A and B. Solvent A was HPLC grade water with 0.1% (v/v) formic acid, and solvent B was HPLC grade acetonitrile 80% (v/v) with 0.1% (v/v) formic acid. Separations were performed by applying a linear gradient of 3.8% to 40% solvent B over 90 min at 300 nL/min followed by a washing step (5 min at 99% solvent B) and an equilibration step (15 min at 3.8% solvent B).

The Q Exactive mass spectrometer was operated in data dependent positive (ESI+) mode to automatically switch between full scan MS and MS/MS acquisition. Survey full scan MS spectra (*m/z* 300-2000) were acquired in the Orbitrap with 70,000 resolution (*m/*z 200) after accumulation of ions to 1x10^6^ target value based on predictive automatic gain control (AGC) values from the previous full scan. Dynamic exclusion was set to 20s. The 10 most intense multiply charged ions (*z* ≥ 2) were sequentially isolated and fragmented in the octopole collision cell by higher energy collisional dissociation (HCD) with a fixed injection time of 100 ms and 35,000 resolution. Typical mass spectrometric conditions were as follows: spray voltage, 2.1 kV, no sheath or auxillary gas flow; heated capillary temperature, 280°C; normalised HCD collision energy 30%. The MS/MS ion selection threshold was set to 1 x 10^5^ counts and a 2 *m/z* isolation width was set.

Raw data files were uploaded into Proteome Discoverer 1.3 and searched against the human reviewed UniProt database using the Mascot search engine (version 2.4.1). A precursor ion tolerance of 10ppm and a fragment ion tolerance of 0.01Da were used with carbamidomethyl cysteine set as a fixed modification and oxidation of methionine as a variable modification. A quantification workflow using the area of the top 3 most intense peptides of each protein relative to the top 3 area of yeast enolase (*Saccharomyces cerevisiae*) protein standard was used to approximate a label-free quantification of each protein.

**Detailed protocols for immunofluorescence and immunohistochemistry:**

**Immunofluorescence**

| **Name** | **Species** | **Supplier** | **Antigen Retrieval** | **Concentration** |
| --- | --- | --- | --- | --- |
| CTNNNB1 Clone 6F9 | Mouse | Sigma | Citrate pH6 or Tris-EDTA pH9 | 1:300 |
| \| BCL11B/CTIP2  Clone 25B6 (ab18465) \| \| --- \| | Rat | Abcam | Citrate pH6 | 1:250 |
| TP63 Clone 4A4 (ab735) | Mouse | Abcam | Tris-EDTA pH9 | 1:250 |
| P21 Clone M19 (sc-471) | Rabbit | Santa Cruz | Tris-EDTA pH9 | 1:400 |
| EDAR Clone E-19 (sc-15289) | Goat | Santa Cruz | Tris-EDTA pH9 | 1:100 |
| EDA Clone C-17 (sc-18927) | Goat | Santa Cruz | Tris-EDTA pH9 | 1:200 |
| pERK1/2 #9101 | Rabbit | Cell Signalling | Citrate pH6 | 1:250 |
| Ki67 (ab42170) | Rabbit | Abcam | Tris-EDTA pH9 | 1:100 |
| pSMAD3 (ab52903) | Rabbit | Abcam | Tris-EDTA pH9 | 1:100 |
| pSMAD1,5,8/9 (#9511) | Rabbit | Cell Signalling | Tris-EDTA pH9 | 1:200 |
| CASPASE-3 antibody #9664 | Rabbit | Cell Signalling | Tris-EDTA pH9 | 1:300 |
| APCDD1L (NBP2-49502PEP) | Rabbit | Novus Biologicals | Citrate pH6 | 1:2500 |

- 1. **3,3′-Diaminobenzidine (DAB) Immunohistochemistry**

The following were performed on Leica BondMax® automated immunohistochemistry machine as follows:

| **Name** | **Species** | **Supplier** | **Detection kit** | **Concentration** |
| --- | --- | --- | --- | --- |
| CD68 (PGM1) (clone 514H12, cat PA0273) | Mouse | LEICA | Bond-Max Protocol F | 1:100 |
| CD4 (clone 4B12, cat PA0368) | Mouse | LEICA | Bond-Max Protocol F | Pre-diluted |
| CD3 (clone LN10, cat PA0553) | Mouse | LEICA | Bond-Max Protocol F | Pre-diluted |
| CD8 (clone 4B11, cat PA0183) | Mouse | LEICA | Bond-Max Protocol F | Pre-diluted |
| GFAP (cat 20334) | Rabbit | DAKO | Bond-Max Protocol F | 1:2000 |
| β CATENIN (clone Bcat-1,code M3539) | Mouse | DAKO | Bond-Max Protocol F | 1:100 |
| pERK1/2 (197G2) | Rabbit | Cell Signalling | Bond-Max  Protocol F | 1:50 |
| CCL2 (HPA019163) | Rabbit | Sigma | Bond-Max Protocol F | 1:100 |
| 4.16H1 anti-IDO-1 antibody | Rabbit | Dr Van den Eynde | Cancer Immunol Res. 2015;3:161-72 | 1:1000 |

**SUPPLEMENTARY FIGURE LEGENDS**

Suppl. Fig. 1. Clustering of the human samples using for RNA Seq and module preservation with the dataset of Gump et al. (2016).

**a** Hierarchical clustering of samples used in this study showing the separation of controls from tumours. Note that samples JA002 and JA004, both from the same patient, group together and that JA051, the only adult ACP sample, groups with other paediatric ACP samples. The heatmap is further annotated with sample information. Blue indicates the type of samples (e.g whether the sample is NFPA or fetal pituitary) or the presence of a trait (e.g. abundance of glial tissue or wet keratin). For instance, tumours JA005, JA011, JA010, JA020, JA029, JA002, JA004 and JA051 contain abundant reactive glia tissue whilst all the other tumours did not contain glial tissue in the frozen sections (white) or could not be assessed usually due to freezing damaged (grey). Likewise, wet keratin was a prominent feature in tumours JA029, JA002, JA008, JA026, JA056, JA009, JA023 and JA053 whilst less prominent is JA005, JA011 and JA004. The other tumours did not contain any wet keratin in the frozen section (white) or could not be assessed (grey). The assessment of tumour content and *CTNNB1* mutation allele frequency is represented in red colour, the higher the value the more intense the red. For instance, JA009 shows a high tumour content and *CTNNB1* mutation allele frequency.

**b** Multi-dimensional scaling of the expression pattern 2963 matched genes from Gump *et al*, 2016 annotated with the colors of module identified in our cohort. **c** Assessment of module preservation in the dataset of Gump *et al*., 2016. Modules are plotted by colour. Values over 10 (green line) indicate strong evidence of module is presrvation. Values between 2 (blue line) and 10 indicate weak to moderate evidence of preservation. The pink, magenta, brown, blue and green are the most preserved modules. The cyan module is the least preserved. This represents cell cycle genes expressed by proliferating fetal pituitary tissue. As the normal pituitary tissue analyzed in Gump *et al*., 2016 is adult, it is expected that this module would be the least preserved.

Suppl. Fig. 2: Heatmaps for the brown, black, dark-grey and magenta gene expression modules obtained from *in silico* analysis.

Heatmaps of gene expression for each module. Gene expression is scaled by row across the heatmap. Yellow indicates high expression, red low expression. Heatmaps are annotated with samples’ names and a brief summary of significant characteristics. The brown module (a) shows genes expressed only in tumours, the black (b) predominantly in those tumours with wet keratin and the dark-grey (c) in most tumours and controls with the exception of JA005, JA011 and JA038. The magenta module genes (d) are expressed minimally in controls and tumours with high tumour content. Pits: pituitaries.

Suppl. Fig. 3: Heatmaps for the blue, dark-turquoise, green and light-yellow gene expression modules obtained from *in silico* analysis.

Heatmaps of gene expression for each module. Gene expression is scaled by row across the heatmap. Yellow indicates high expression, red low expression. Heatmaps are annotated with samples’ names and brief summary of significant characteristics. The blue, dark-turquoise and green module genes (a-c) are expressed in tumour samples with glial tissue and in some controls. The light-yellow module genes (d) are highly expressed in fetal pituitaries and a subset of tumours (JA026, JA056, JA023), correlating with those with the most wet keratin.

Suppl. Fig. 4: Heatmaps for the cyan and light-green gene expression modules obtained from *in silico* analysis.

Heatmaps of gene expression for each module. Gene expression is scaled by row across the heatmap. Yellow indicates high expression, red low expression. Heatmaps are annotated with samples’ names and brief summary of significant characteristics. The pink, cyan and light-green modules are variably expressed in controls. The grey module contains genes that do not fall into modules. It includes a small subset of genes relating to sex determination (e.g. *XIST*, *TSIX*, *PRKY*, *ZFY*), information regarding patient sex was only available in a small number of cases and where available was consistent with expression of these genes.

Suppl. Fig. 5: Expression analysis of APCDD1L in human ACP.

Double immunofluorescence against APCDD1L and β-catenin on histological sections of human ACP. Note the expression of APCDD1L in the tumour tissue, mostly in the β-catenin-accumulating clusters (C), as well as in glia cell adjacent to the tumour (G). However, no APCDD1L staining is observed in the vast majority of the glial cells further away from the tumour. The bottom panel depicts an area of the section with only glial cells and no tumour epithelial cells, which is devoid of any APCDD1L staining. Scale bars: 100 μm.

Suppl. Fig. 6: Expression of *TP63*, *APCDD1L* and *BCL11B* correlates with the *CTNNB1* mutation frequency.

Each dot represents a tumour sample from **Suppl. Table 1 (Online resource 2**).

Suppl. Fig. 7: Expression of ligands, receptors and downstream signals in clusters, palisading epithelium and glial reactive tissue.

**a** Plots of normalised counts of two ACP samples for *SHH*, *FGF3*, *TGFB1* and *BMP4* showing higher expression in the clusters (C) relative to palisading epithelium (PE) and glial reactive tissue (G). Normalised counts are on a log2 scale. Colours indicate case, note duplicates for C and PE for case JA004b. **b** Heatmaps of normalised counts for specific members of the FGF, TGFβ, BMP and WNT families of secreted factors and their receptors.

Suppl. Fig. 8: Proteome analysis of cystic fluid.

**a** SDS-PAGE analysis of cystic fluid revealed a predominance of a single protein at 66kDa, predicted to be serum albumin and confirmed by proteomics analysis (**Suppl. Table 7 (Online Resource 9)**). After the fluid was equalised using a bead-bound combinatorial peptide library, the relative intensities of individual proteins were considerably more even, and many more proteins were visible on the gel. Cystic fluid treated this way was used for subsequent proteome analysis of six patient samples.

**b** Six samples of cystic fluid were analysed by ‘discovery’ proteomics after equalisation using Proteominer beads. A total of 461 proteins were identified in all six samples. Label free quantification, using yeast enolase as a standard, revealed a wide range of abundances, ranging from 5-10 pmol of abundant proteins, such as apolipoproteins, to less than 0.1 fmol/100μL for the lowest abundant proteins. A matrix plot of the log(protein abundance) for all samples indicated a broad similarity between all six samples

**c** Overall proteome profile of equalised cystic fluid samples. The data for all six patient samples were combined, and the log of the average protein abundance (expressed as pmol protein in 100μL equalised cystic fluid) was plotted for 461 proteins.

**d** The 461 proteins in the ACP fluid were analysed for enriched gene ontogeny terms using GOrilla (<http://cbl-gorilla.cs.technion.ac.il/)> and visualised for high scoring terms using Revigo (<http://revigo.irb.hr/)> to reduce and visualise gene ontology terms. High scoring processes are labelled.

**References**

1 Binns D, Dimmer E, Huntley R, Barrell D, O'Donovan C, Apweiler R (2009) QuickGO: a web-based tool for Gene Ontology searching. Bioinformatics 25: 3045-3046 Doi 10.1093/bioinformatics/btp536

2 Buslei R, Holsken A, Hofmann B, Kreutzer J, Siebzehnrubl F, Hans V, Oppel F, Buchfelder M, Fahlbusch R, Blumcke I (2007) Nuclear beta-catenin accumulation associates with epithelial morphogenesis in craniopharyngiomas. Acta Neuropathol 113: 585-590 Doi 10.1007/s00401-006-0184-3 [doi]

3 Chevillard G, Derjuga A, Devost D, Zingg HH, Blank V (2007) Identification of interleukin-1beta regulated genes in uterine smooth muscle cells. Reproduction (Cambridge, England) 134: 811-822 Doi 10.1530/rep-07-0289

4 Falcon S, Gentleman R (2007) Using GOstats to test gene lists for GO term association. Bioinformatics 23: 257-258 Doi 10.1093/bioinformatics/btl567

5 Gaston-Massuet C, Andoniadou CL, Signore M, Jayakody SA, Charolidi N, Kyeyune R, Vernay B, Jacques TS, Taketo MM, Le Tissier Pet al (2011) Increased Wingless (Wnt) signaling in pituitary progenitor/stem cells gives rise to pituitary tumors in mice and humans. Proc Natl Acad Sci U S A 108: 11482-11487 Doi 10.1073/pnas.1101553108

6 Gump JM, Donson AM, Birks DK, Amani VM, Rao KK, Griesinger AM, Kleinschmidt-DeMasters BK, Johnston JM, Anderson RC, Rosenfeld Aet al (2015) Identification of targets for rational pharmacological therapy in childhood craniopharyngioma. Acta Neuropathol Commun 3: 30 Doi 10.1186/s40478-015-0211-5

7 Helsinki DBPoUo (1996-2007) Gene expression in tooth database <http://bite-it.helsinki.fi> Developmental Biology Programme of University of Helsinki. Accessed 19th July 2016

8 Horvath S, Zhang B, Carlson M, Lu KV, Zhu S, Felciano RM, Laurance MF, Zhao W, Qi S, Chen Zet al (2006) Analysis of oncogenic signaling networks in glioblastoma identifies ASPM as a molecular target. Proc Natl Acad Sci U S A 103: 17402-17407 Doi 10.1073/pnas.0608396103

9 Jura J, Wegrzyn P, Korostynski M, Guzik K, Oczko-Wojciechowska M, Jarzab M, Kowalska M, Piechota M, Przewlocki R, Koj A (2008) Identification of interleukin-1 and interleukin-6-responsive genes in human monocyte-derived macrophages using microarrays. Biochim Biophys Acta 1779: 383-389 Doi 10.1016/j.bbagrm.2008.04.006

10 Langfelder P, Horvath S (2008) WGCNA: an R package for weighted correlation network analysis. BMC Bioinformatics 9: 559 Doi 10.1186/1471-2105-9-559

11 Langfelder P, Luo R, Oldham MC, Horvath S (2011) Is my network module preserved and reproducible? PLoS Comput Biol 7: e1001057 Doi 10.1371/journal.pcbi.1001057

12 Liao Y, Smyth GK, Shi W (2013) The Subread aligner: fast, accurate and scalable read mapping by seed-and-vote. Nucleic Acids Res 41: e108 Doi 10.1093/nar/gkt214

13 Love MI, Huber W, Anders S (2014) Moderated estimation of fold change and dispersion for RNA-seq data with DESeq2. Genome Biol 15: 550 Doi 10.1186/s13059-014-0550-8

14 Ritchie ME, Phipson B, Wu D, Hu Y, Law CW, Shi W, Smyth GK (2015) limma powers differential expression analyses for RNA-sequencing and microarray studies. Nucleic Acids Res 43: e47 Doi 10.1093/nar/gkv007

15 Stone TJ, Keeley A, Virasami A, Harkness W, Tisdall M, Izquierdo Delgado E, Gutteridge A, Brooks T, Kristiansen M, Chalker Jet al (2018) Comprehensive molecular characterisation of epilepsy-associated glioneuronal tumours. Acta Neuropathol 135: 115-129 Doi 10.1007/s00401-017-1773-z

16 Subramanian A, Tamayo P, Mootha VK, Mukherjee S, Ebert BL, Gillette MA, Paulovich A, Pomeroy SL, Golub TR, Lander ESet al (2005) Gene set enrichment analysis: a knowledge-based approach for interpreting genome-wide expression profiles. Proc Natl Acad Sci U S A 102: 15545-15550 Doi 10.1073/pnas.0506580102

17 Sulkava M, Raitoharju E, Levula M, Seppala I, Lyytikainen LP, Mennander A, Jarvinen O, Zeitlin R, Salenius JP, Illig Tet al (2017) Differentially expressed genes and canonical pathway expression in human atherosclerotic plaques - Tampere Vascular Study. Sci Rep 7: 41483 Doi 10.1038/srep41483

18 Vincenti MP, Brinckerhoff CE (2001) Early response genes induced in chondrocytes stimulated with the inflammatory cytokine interleukin-1beta. Arthritis research 3: 381-388 Doi 10.1186/ar331

19 Yoshihara K, Shahmoradgoli M, Martinez E, Vegesna R, Kim H, Torres-Garcia W, Trevino V, Shen H, Laird PW, Levine DAet al (2013) Inferring tumour purity and stromal and immune cell admixture from expression data. Nat Commun 4: 2612 Doi 10.1038/ncomms3612

20 Young MD, Wakefield MJ, Smyth GK, Oshlack A (2010) Gene ontology analysis for RNA-seq: accounting for selection bias. Genome Biol 11: R14 Doi 10.1186/gb-2010-11-2-r14

21 Zhang B, Horvath S (2005) A general framework for weighted gene co-expression network analysis. Stat Appl Genet Mol Biol 4: Article17 Doi 10.2202/1544-6115.1128

22 Zhang Y, Chen K, Sloan SA, Bennett ML, Scholze AR, O'Keeffe S, Phatnani HP, Guarnieri P, Caneda C, Ruderisch Net al (2014) An RNA-sequencing transcriptome and splicing database of glia, neurons, and vascular cells of the cerebral cortex. J Neurosci 34: 11929-11947 Doi 10.1523/JNEUROSCI.1860-14.2014
