# Supplementary material for: Tumour compartment transcriptomics demonstrates the activation of inflammatory and odontogenic programmes in human adamantinomatous craniopharyngioma and identifies the MAPK/ERK pathway as a novel therapeutic target
Source: Acta Neuropathol. 2018 Mar 14;135(5):757–77. doi: 10.1007/s00401-018-1830-2 (PMC5904225; doi:10.1007/s00401-018-1830-2)
Supplement: Supplementary file 6 — Supplementary material 6 (DOCX 134 kb) [file 401_2018_1830_MOESM6_ESM.docx]

**Supplementary Table 5 (Online Resource 7)**

**Tumour compartment transcriptomics demonstrate the activation of inflammatory and odontogenic programmes in human adamantinomatous craniopharyngioma and identify the MAPK/ERK pathway as novel therapeutic target**

 John R. Apps^1,2,*^, Gabriela Carreno^1^, Jose Mario Gonzalez-Meljem^1,3^, Scott Haston^1^, Romain Guiho^1^, Julie E. Cooper^1^, Saba Manshaei^1^, Nital Jani^4^, Annett Hölsken^5^, Benedetta Pettorini^6^, Robert J. Beynon^7^, Deborah M. Simpson^7^, Helen C. Fraser^1^, Ying Hong^8^, Shirleen Hallang^9^, Thomas J. Stone^1,2^, Alex Virasami^2^, Andrew M. Donson^10^, David Jones^11^, Kristian Aquilina^12^, Helen Spoudeas^13^, Abhijit R. Joshi^14^, Richard Grundy^15^, Lisa CD Storer^15^, Márta Korbonits^16^, David A. Hilton^17^, Kyoko Tossell^18^, Selvam Thavaraj^19^, Mark A. Ungless^18^, Jesus Gil^18^, Rolf Buslei^5,20^, Todd Hankinson^10^, Darren Hargrave^21^, Colin Goding^22^, Cynthia L. Andoniadou^23,24^, Paul Brogan^8,25^, Thomas S. Jacques^1,2^, Hywel J. Williams^4^ and Juan Pedro Martinez-Barbera^1,*^

1) Developmental Biology and Cancer Programme, Birth Defects Research Centre, UCL Great Ormond Street Institute of Child Health, University College London, London, UK

2) Histopathology Department, Great Ormond Street Hospital NHS Trust, London, UK

3) Basic Research Department, National Institute of Geriatrics, Mexico City, Mexico

4) Centre for Translational Omics - GOSgene, Genetics and Genomic Medicine Programme, UCL Institute of Child Health, University College London, London, UK

5) Department of Neuropathology, Friedrich-Alexander University Erlangen-Nürnberg (FAU), Erlangen, Germany

6) Alder Hey Children’s Hospital NHS Foundation Trust, Liverpool, UK

7) Centre for Proteome Research, Institute of Integrative Biology, University of Liverpool, UK

8) Infection, Immunity and Inflammation Programme, UCL Great Ormond Street Institute of Child Health, University College London, London, UK

9) Centre for Craniofacial and Regenerative Biology, King's College London, London, UK

10) Department of Pediatrics, University of Colorado Anschutz Medical Campus, Aurora, Colorado

11) German Cancer Research Center (DKFZ), Heidelberg, Germany

12) Neurosurgery Department, Great Ormond Street Hospital NHS Trust, London, UK

13) Endocrinology Department, Great Ormond Street Hospital NHS Trust, London, UK

14) Laboratory Medicine, Royal Victoria Infirmary, Newcastle, UK

15) Children’s Brain Tumour Research Centre, University of Nottingham, Nottingham, UK

16) William Harvey research Institute, Barts and the London School of Medicine and Dentistry, Queen Mary University, London, UK

17) Pathology Department, Plymouth Hospitals NHS Trust, Plymouth, UK

18) MRC London Institute of Medical Sciences, Imperial College London, London, UK

19) Head and Neck Pathology, Dental Institute, King's College London, London, UK

20) Institute of Pathology, Klinikum Sozialstiftung Bamberg, Bamberg, Germany

21) Haematology and Oncology department, Great Ormond Street Hospital NHS Trust, London, UK

22) Ludwig Institute for Cancer Research, Oxford University, Old Road Campus, Headington, Oxford, UK

23) Centre for Craniofacial and Regenerative Biology, King’s College London, Guy’s Hospital, Floor 27 Tower Wing, London, UK

24) Department of Internal Medicine III, Technische Universität Dresden, Fetscherstaße 74, 01307 Dresden, Germany

25) Rheumatology department, Great Ormond Street Hospital NHS Trust, London, UK

* Corresponding authors: John Apps [j.apps@ucl.ac.uk](mailto:j.apps@ucl.ac.uk), JP Martinez-Barbera, [j.martinez-barbera@ucl.ac.uk](mailto:j.martinez-barbera@ucl.ac.uk). +44 (0) 207 905 2821

**Supplementary Table 5**. Differential expression of genes expressed during tooth development. Values higher than 1: higher expression in tumours than control tissues. Values <1 : higher expression in control tissues than tumours

| **Gene** | **Log fold change** | **Fold change** | **Adjusted p value** |
| --- | --- | --- | --- |
| BCL11B | 3.95 | 15.45 | 4.44E-09 |
| MSX2 | 9.96 | 995.99 | 4.19E-48 |
| DLX1 | 7.68 | 205.07 | 3.00E-25 |
| DLX2 | 8.36 | 328.56 | 1.24E-23 |
| DLX3 | 5.99 | 63.56 | 2.09E-11 |
| DLX6 | 6.75 | 107.63 | 9.04E-22 |
| SP6 | 5.39 | 41.93 | 9.40E-10 |
| AMELX | 6.28 | 77.71 | 2.73E-06 |
| AMELY | 3.05 | 8.28 | 0.078 |
| ENAM | 4.61 | 24.42 | 0.00026 |
| AMBN | 8.29 | 312.99 | 5.96E-13 |
| KLK4 | 7.98 | 252.47 | 1.51E-12 |
| MMP20 | 7.25 | 152.22 | 2.07E-08 |
| ODAM | 9.80 | 891.44 | 4.61E-29 |
| EDAR | 8.87 | 467.88 | 1.37E-29 |
| MSX1 | -1.23 | -2.35 | 0.028 |
| DSPP | 0.99 | 1.99 | 0.42 |
